# A review of asymmetric synthetic organic electrochemistry and electrocatalysis: concepts, applications, recent developments and future directions

**DOI:** 10.3762/bjoc.15.264

**Published:** 2019-11-13

**Authors:** Munmun Ghosh, Valmik S Shinde, Magnus Rueping

**Affiliations:** 1 KAUST Catalysis Center (KCC), King Abdullah University of Science and Technology, Thuwal 23955-6900, Saudi Arabia

**Keywords:** chiral auxiliary, chiral catalyst, chiral electrode, chiral electrolyte, chiral mediator, electroorganic chemistry

## Abstract

The direct exploitation of ‘electrons’ as reagents in synthetic organic transformations is on the verge of a renaissance by virtue of its greenness, sustainability, atom economy, step economy and inherent safety. Achieving stereocontrol in such organic electrochemical reactions remains a major synthetic challenge and hence demands great expertise. This review provides a comprehensive discussion of the details of stereoselective organic electrochemical reactions along with the synthetic accomplishments achieved with these methods.

## Introduction

Electric current-assisted exchange of electrons between an electrode and an organic substrate, resulting in desired redox transformation of the substrate via the intermediacy of a highly reactive electrogenerated reagent (radical, ion or ionic radical), is generally termed ‘organic electrochemical reactions’. Amidst tremendous effort by synthetic organic chemists towards reaching targets selectively from readily available starting materials using low cost, nontoxic reagents and solvents while maintaining high atom and step economy and minimizing waste production with respect to safety standards, electroorganic chemistry (EOC) stands out as a potential greener and sustainable alternative to traditional redox protocols [1–4]. Starting from its inception in 1800, EOC has undergone a series of advances with respect to the design of electrochemical cells; the nature of the electrode materials; the applied current or potential; the available redox mediators, electroauxiliaries, supporting electrolyte, types of catalysts; and many other controlling factors [5–7]. The complex history of organic electrosynthesis has been revisited in detail in a number of articles [8–12]. The two sequential reviews from Baran’s group and Waldvogel’s group focused on the advancements achieved in the development of synthetic methods as well as applications of organic electrosynthesis reported since 2000 [13–14]. Moreover, in his recent article, Moeller have nicely demonstrated that both physical and organic chemistry are indispensable for exploiting electroorganic reactions to their fullest extent [15].

Asymmetric electrochemical synthesis refers to electroorganic reactions resulting in the introduction of one or more new elements of chirality into a target compound. The induction of asymmetry into achiral substrates through electrochemical methods can be achieved by using suitable chiral sources [16]. Several groups of organic chemists have been involved in establishing and improving the effectiveness of chiral inductors for electrosynthesis; as a result, a number of reports have been published in this important field of synthetic organic chemistry. In this review, we aim to focus on methods for achieving stereocontrol in synthetic organic electrochemistry via a systematic description of the reported literature on chiral inductors, followed by their applications in the synthesis of natural products and bioactive compounds including late-stage functionalizations. Although this field has been periodically reviewed over the last few decades, a single article addressing all attempts to date will be useful for organic chemists working in this field and for those who would like to obtain an overview of the current state of the art of asymmetric electrocatalytic reactions.

## Review

### Classification and systematic description of chiral inductors

**Figure 1:**
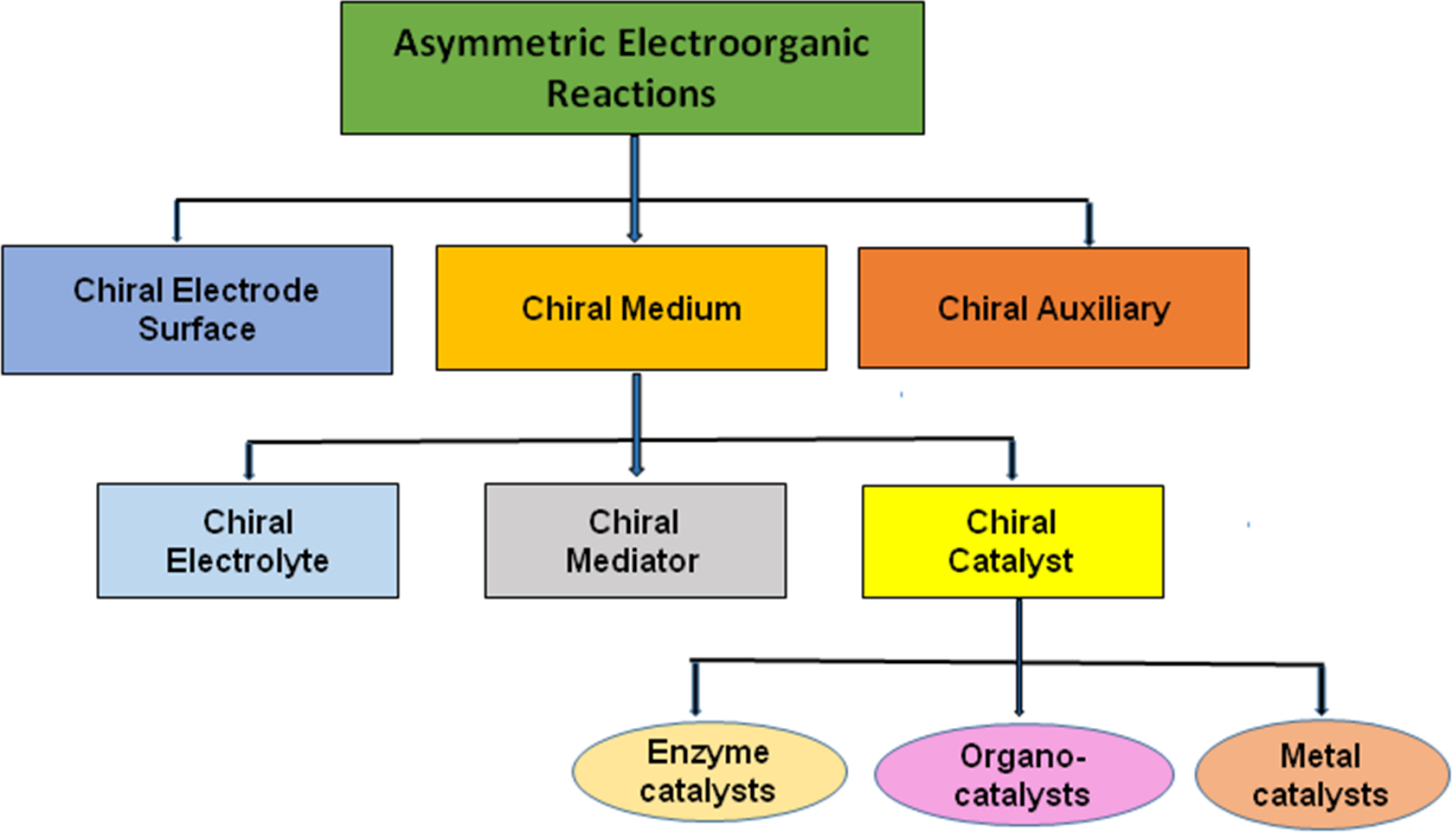
General classification of asymmetric electroorganic reactions.

Transformations of achiral organic substrates into chiral products by electrochemical synthetic methods require the active participation of an external source of chirality. Organic chemists working in this area have established a number of asymmetric electrochemical inductors. After a thorough comprehension of the available literature, we propose to classify the chiral electrochemical inductors into three broad categories: chiral electrodes, chiral media and chiral auxiliaries [17]. We have summarized the background and recent advances and arranged this article to provide a systematic description of electrochemical reactions in which chirality has been induced using these three sources (Figure 1).

### Chemically modified chiral electrodes

#### Electrochemical asymmetric reductions using chiral electrodes

**Scheme 1:**
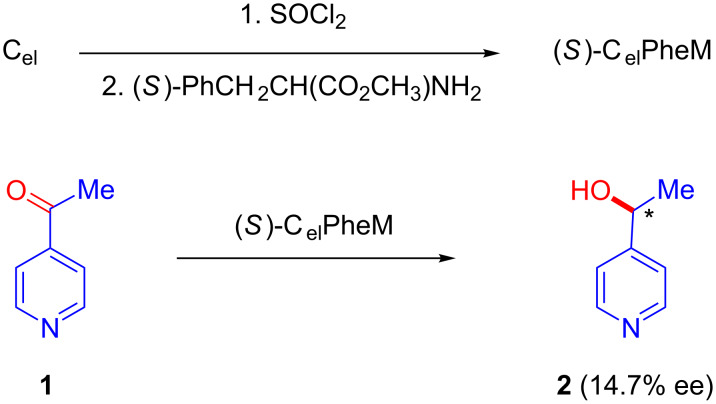
Asymmetric reduction of 4-acetylpyridine using a modified graphite cathode.

Electrodes with chiral surfaces have long been prepared through the adsorption of chiral active auxiliaries onto the surface of the metallic electrode. In such cases, the chiral electrode becomes an inherent part of the electrochemical cell and serves as a heterogeneous catalyst. A recent article by Wattanakit described several approaches for the development of chiral metal electrodes for enantioselective recognition and asymmetric synthesis developed over the past decade [18]. Concurrent with pioneering works in the field of asymmetric induction in electrochemical reduction on chiral mercury cathodes in the presence of chiral inductors, including tertiary amines, optically active proteins, and alkaloids [19–20], in 1975, Miller’s group reported the first example of the modification of electrodes via covalent binding [21]. They chemically modified air-oxidized graphite electrodes via treatment with thionyl chloride followed by derivatization with (*S*)-(−)-phenylalanine methyl ester. The applicability of this modified electrode [(*S*)-C_el_PheM] was tested by using it as a cathode for the electrochemical reduction of 4-acetylpyridine (**1**). GC analysis of the product indicated the formation of the corresponding alcohol **2** with 14.7% optical yield [21]. Control experiments confirmed that the modified electrodes served as chiral inductors (Scheme 1).

**Scheme 2:**
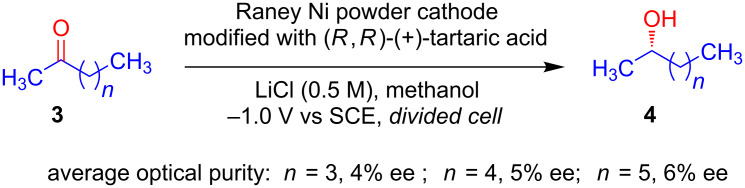
Asymmetric hydrogenation of ketones using Raney nickel powder electrodes modified with optically active tartaric acid.

In 1982, Fujihira and Osa reported the modification of Raney nickel powder electrodes with optically active tartaric acid and used these electrodes for the asymmetric hydrogenation of ketones [22]. They showed that upon reduction with Raney nickel powder electrodes modified with (*R*,*R*)-(+)-tartaric acid, 2-hexanone **3** was converted to (*S*)-(+)-2-hexanol **4** with an average optical purity of 4%, whereas Raney nickel powder electrodes modified with (*S*,*S*)-(−)-tartaric acid produced (*R*)-(−)-2-hexanol with almost the same optical purity. Similar results were observed when 2-heptanone and 2-octanone were used as substrates (Scheme 2).

**Scheme 3:**
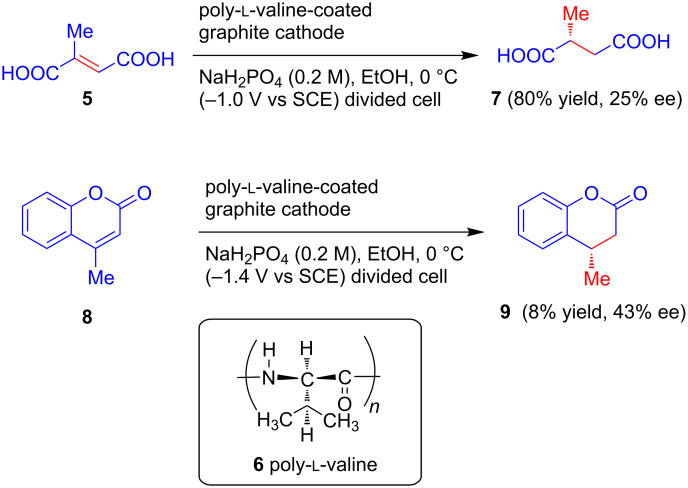
Asymmetric reduction of prochiral activated olefins with a poly-ʟ-valine-coated graphite cathode.

**Scheme 4:**
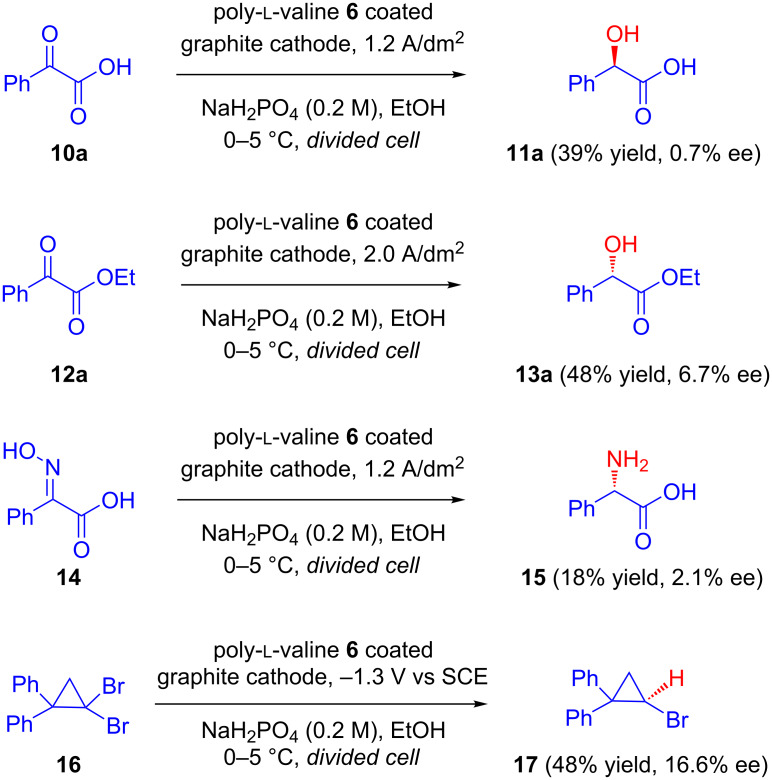
Asymmetric reduction of prochiral carbonyl compounds, oximes and *gem*-dibromides on a poly-ʟ-valine-coated graphite cathode.

The above results confirm that chemically modified chiral electrodes are a useful platform for asymmetric induction because they require a small amount of inducing agents. However, poor coverage of the electrode surface with the chiral species was soon noted as one of the major disadvantages of this method, and chemists started developing polymer-coated electrodes as an alternative. In 1983, Nonaka et al. explored the applicability of a poly-ʟ-valine **6** coated graphite cathode for asymmetric electrochemical reductions in two consecutive reports. In the first communication, they carried out the asymmetric reduction of prochiral activated olefins **5** and **8**, affording **7** and **9** with optical yields of 25% and 43%, respectively (Scheme 3) [23]. In the second report, the same electrode was applied for the asymmetric reduction of prochiral carbonyl compounds **10a** and **12a**, oximes **14** and *gem*-dibromo compounds **16** (Scheme 4). Among all of these the highest optical yield (16.6%) was obtained in the reduction of *gem*-dibromide substrates **16** to corresponding bromo-cyclopropane **17** [24]. According to the authors, the passage of larger amounts of charge to facilitate product isolation might be responsible for the observed lower asymmetric yields.

**Scheme 5:**
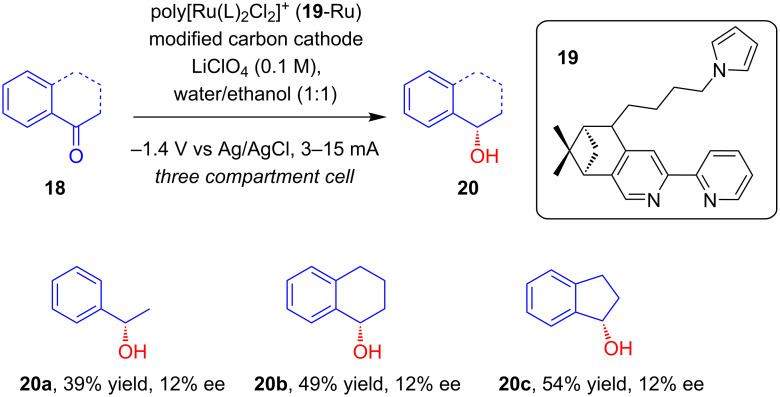
Asymmetric hydrogenation of prochiral ketones with poly[Ru^III^(L)_2_Cl_2_]^+^-modified carbon felt cathodes.

In 1998, Moutet’s group reported for the first time that transition metal complexes with chiral ligands can be used for the chemical modification of electrodes to achieve asymmetric induction in electrosynthesis [25–26]. They showed that carbon electrodes modified with poly[Ru^III^(L)_2_Cl_2_]^+^ (generated from the electropolymerization of [Ru^III^(L)_2_Cl_2_]^+^ complexes **19** on the carbon surface) can effectively catalyze the enantioselective hydrogenation of prochiral ketones **18**. The prochiral ketones such as acetophenone, 1-tetralone and 1-indanone were reduced to their corresponding alcohols **20a**, **20b** and **20c**, respectively, with moderate optical yields, with formation of (*S*) as major enantiomer (Scheme 5).

**Scheme 6:**
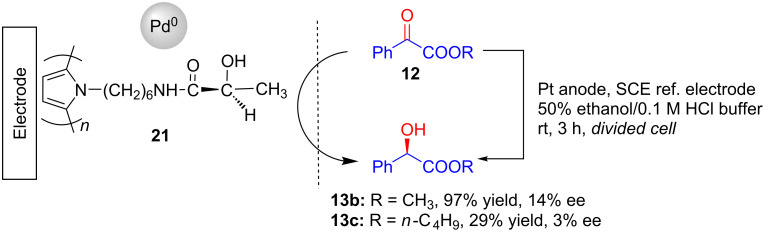
Asymmetric hydrogenation of α-keto esters using chiral polypyrrole film-coated cathode incorporated with palladium metal.

After a long gap, in 2006, Takano and Seki prepared a PLPy(Pd) electrode coated with a chiral poly-(*N*-substituted pyrrole) film having ʟ-(+)-lactic acid moieties as the optically active groups and further incorporated with palladium metal [27]. Thus prepared electrodes **21** were used for the electrocatalytic hydrogenation of α-keto esters **12** to afford corresponding hydroxy esters **13b** and **13c** with appreciable enantioselectivities (Scheme 6).

**Scheme 7:**
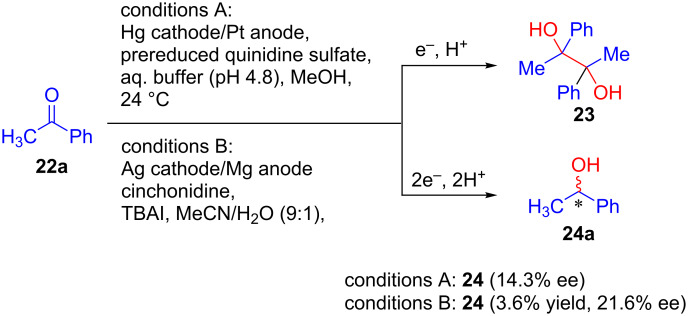
Quinidine and cinchonidine alkaloid-induced asymmetric electroreduction of acetophenone.

Following the pioneering works by Grimshaw et al. [28] in 1973, Gileadi and co-workers showed that the addition of quinidine or related alkaloids to the reaction mixture during the electroreduction of acetophenone on a mercury cathode induces optical activity in the product (alcohol **24a**, Scheme 7) [29]. Additionally, pinacol (**23**) was also obtained via dimerization along with chiral alcohols. The same method was reinvestigated by Wang and Lu in 2013 using a silver cathode in an undivided cell in the presence of cinchonidine alkaloid as the source of chirality [30]. They modified the electrolysis conditions using a mixture of CH_3_CN/H_2_O and tetraethylammonium iodide as the supporting electrolyte and achieved a slight improvement in the enantiomeric excess (Scheme 7, conditions B).

**Scheme 8:**
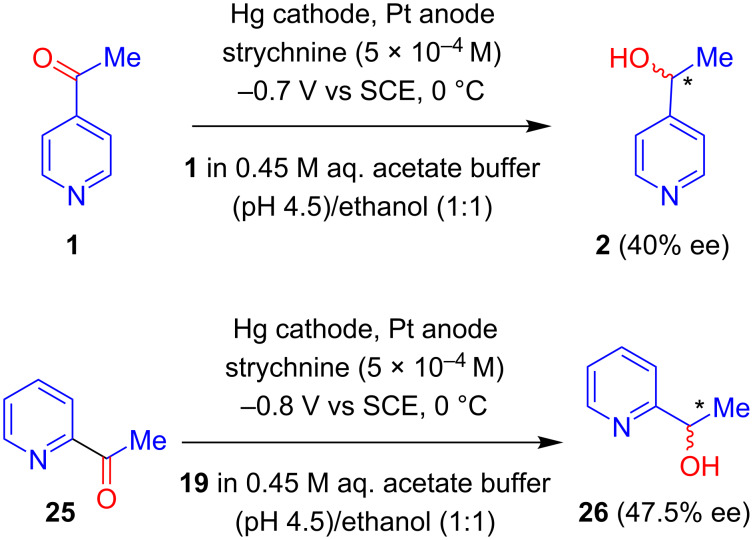
Asymmetric electroreduction of 4- and 2-acetylpyridines at a mercury cathode in the presence of a catalytic amount of strychnine.

Based on a strychnine alkaloid-induced asymmetric electroreduction, Kopilov, Kariv and Miller showed that 4- and 2-acetylpyridines (**1** and **25**) could be reduced to the corresponding pyridylethanols (**2** and **26**, respectively) in presence of a catalytic amount of the alkaloid on a mercury cathode. Notably, the highest optical yields were achieved using strychnine (Scheme 8) [31].

**Scheme 9:**
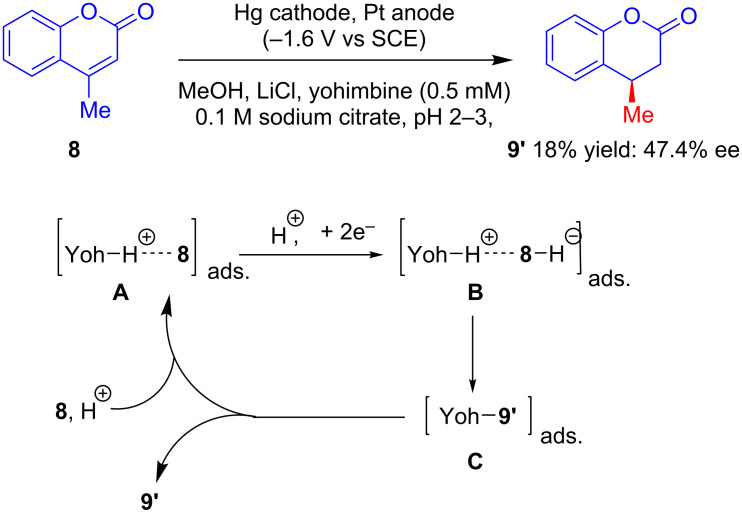
Enantioselective reduction of 4-methylcoumarin in the presence of catalytic yohimbine.

Similarly, by applying modified electrolytic conditions, in 1993, Schoo and Schäfer increased the enantioselectivity of the alkaloid-catalyzed enantioselective electroreduction of 4-methylcoumarin (**8**) relative to that reported previously by Grimshaw’s group in 1970 [28] (the optical yield of **9'** was increased from 17% to 47.4% at the expense of a decrease in the chemical yield from 57% to 18%). According to the authors, protonated alkaloid Yoh-H^+^ initially interacts with **8** to give complex **A,** which after 2e^−^ reduction forms chiral ion pair **B**. The intermediate then undergoes enantioselective protonation by Yoh-H^+^ to generate Yoh-bound **9'** (complex **C**). Subsequent release of the reduced product **9'** and reprotonation of yohimbine complete the cycle (Scheme 9) [32–33].

**Scheme 10:**
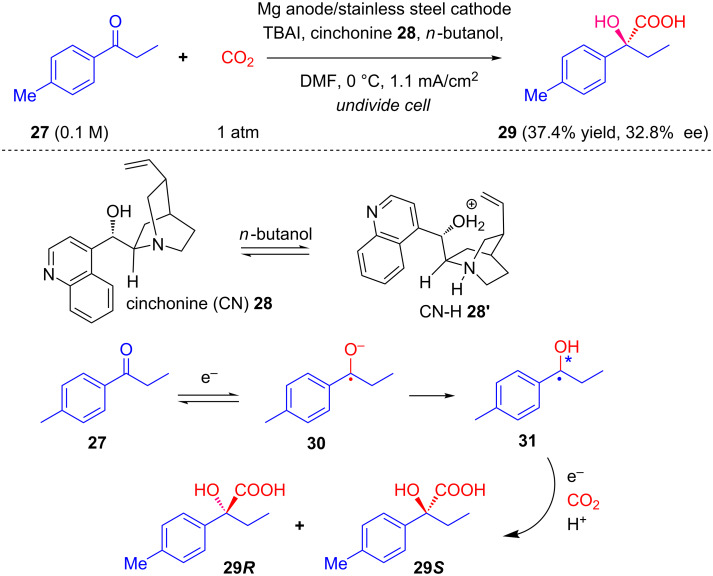
Cinchonine-induced asymmetric electrocarboxylation of 4-methylpropiophenone.

In a continuation of their previous work [34], Wang and Lu investigated the enantioselective electrocarboxylation of 4-methylpropiophenone (**27**) on a stainless steel cathode in which the enantiodiscrimination was controlled by the nucleophilic quinuclidine nitrogen atom and the -OH group of the inductor alkaloid [35]. Mechanistically, cinchonine (CN) abstracts a proton from the cocatalyst (*n*-butanol), which results in the protonation of the quiniclidine *N*-atom to give CN–H. At the same time, compound **27** undergoes a one-electron reduction at the cathode to afford radical anion **30,** which is protonated by CN–H and generates **31**. This results in the installation of an oxygen atom on one face, promoting the addition of CO_2_ from the other face, resulting in enantioenriched carboxylated product **29** after acidification (Scheme 10). The authors described similar results involving the asymmetric electrocarboxylation of other prochiral aromatic ketones in a following publication [36].

**Scheme 11:**
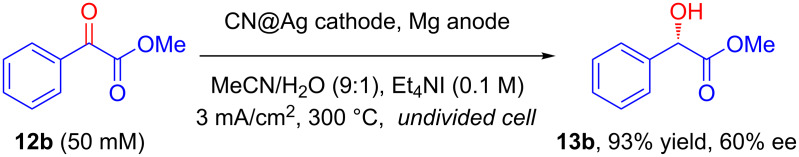
Enantioselective hydrogenation of methyl benzoylformate using an alkaloid entrapped silver cathode.

**Scheme 12:**
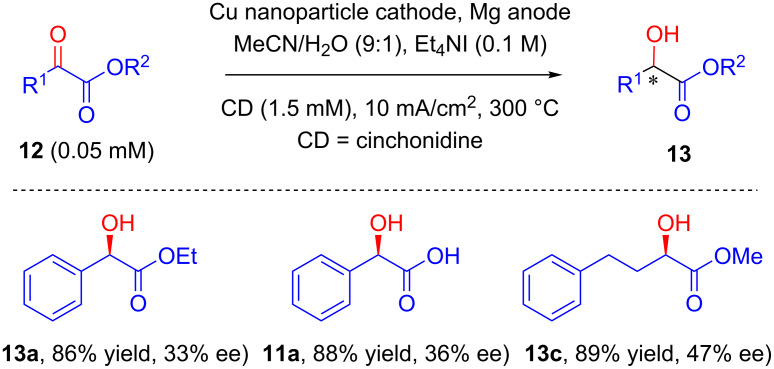
Alkaloid-induced enantioselective hydrogenation using a Cu nanoparticle cathode.

The same group, in 2014, published two concomitant reports on the synthesis of metallo-organic hybrid materials by means of entrapment of alkaloids within silver particles [37–38]. They further used this organically doped metal as cathode for the enantioselective electrohydrogenation of **12b** that resulted in improved yield and enantiomeric purity as compared to previously reported methods (Scheme 11). In another report the same reaction was discovered to be catalyzed by applying copper nanoparticle (CuNP) as a cathode for elelctrohydrogenation of **12** [39]. The asymmetry was induced by addition of certain concentration of alkaloid which was found to get adsorbed on CuNP surface (Scheme 12).

**Scheme 13:**
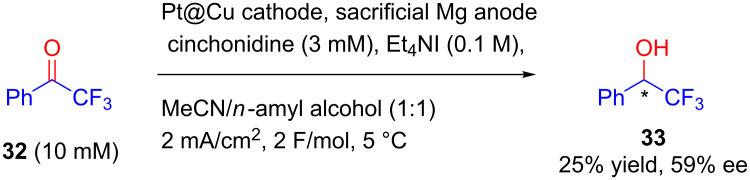
Alkaloid-induced enantioselective hydrogenation of aromatic ketones using a bimetallic Pt@Cu cathode.

In continuation, Wang and Lu, in 2016, developed a ‘copper encapsulated alkaloid composite’ by the entrapment of commercial alkaloids within metallic copper nanoparticles. They compacted this composite as a coin and applied directly as cathode for enantioselective hydrogenation of aromatic ketones. In this case they observed the remarkable stability as well as recyclability of the alkaloid@Cu composite making them reliable for further practical applications [40]. Further, alkaloid-induced asymmetric electrochemical hydrogenation of 2,2,2-trifluoroacetophenone **32** nanoparticles was also developed (Scheme 13). These NPs with low Pt loading were further obtained by reducing platinum precursors on Cu NPs [41].

**Scheme 14:**
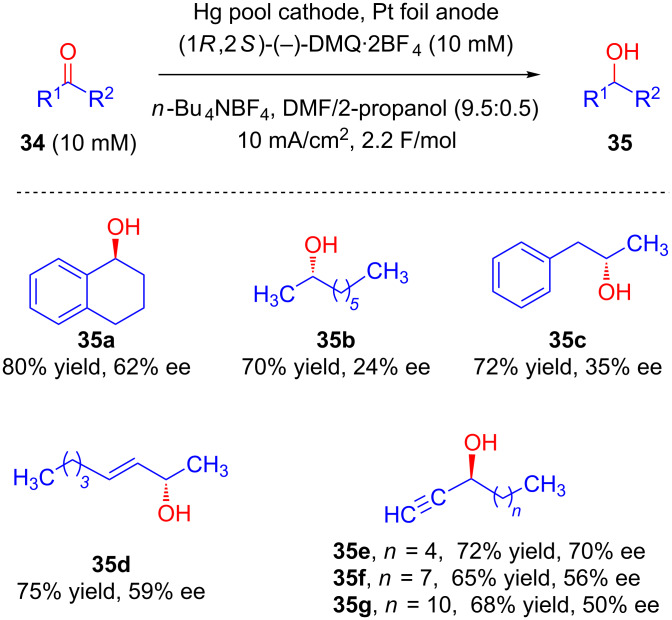
Enantioselective reduction of ketones at mercury cathode using *N*,*N*'-dimethylquininium tetrafluoroborate as chiral inductor.

Yadav’s group, in 2013, described preparative scale enantioselective cathodic reduction of prochiral ketones **34** to their corresponding alcohols **35** at mercury pool in the presence of (−)-*N*,*N*'-dimethylquininium tetrafluoroborate (DMQ·2BF_4_) as chiral inductor using catalytic amounts of (1*R*,2*S*)-(−)-*N*,*N*'-dimethylephedrinium tetrafluoroborate (Scheme 14) [42–43].

**Scheme 15:**
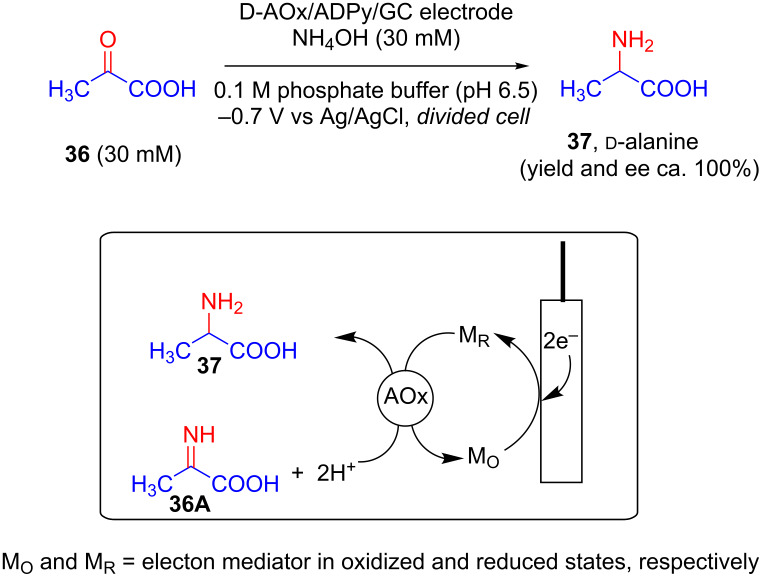
Asymmetric synthesis of an amino acid using an electrode modified with amino acid oxidase and electron mediator.

Kawabata and Yoneyama, in 2000, reported for the first time, a crucial modification of a glassy carbon electrode via chemical immobilization of ᴅ-amino acid oxidase (D-AOx) as enzyme and 1-aminopropyl-1'-methyl-4,4'-dipyridinium iodide (ADPy) as electron mediator and thus prepared electrode D-AOx/ADPy/GC. Electrochemical reduction of pyruvic acid (**36**) using this electrode in a two compartment cell separated by a cation exchange membrane resulted in the production of ᴅ-alanine (**37**) with almost 100% ee (Scheme 15). As per the proposed mechanism, the imino acid **36A** produced from the reaction of acid with NH_4_OH, gets oxidized to the corresponding amino acid via electrochemical reduction of FAD in AOx by electron mediator ADPy. It should be noted that the authors also reported the limitation of this electrochemical method in terms of substrate selectivity of the enzymes used [44].

#### Electrochemical asymmetric oxidation using chirally modified electrodes

Electrochemical oxidation reactions have long served as substantial synthetic tool because of their ability to increase the functionality of organic molecules via reversing the polarity of electron-rich functional groups and thereby generating highly reactive intermediates. Recent advances in anodic cyclization reactions along with their detailed mechanistic rationale have been studied in details in an article published by Moeller’s group [45]. Herein, we present a systematic description of electrooxidation reactions where asymmetry has been induced by means of chiral modification of anodes.

**Scheme 16:**
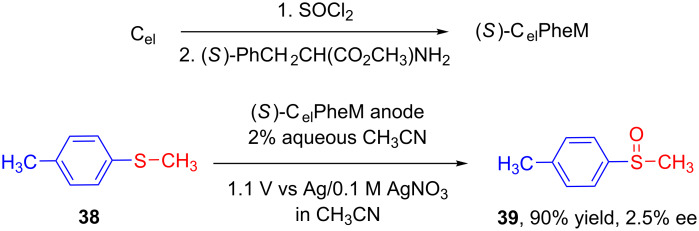
Asymmetric oxidation of *p*-tolyl methyl sulfide using chemically modified graphite anode.

As in case of asymmetric electroreduction, the prime reasonable entry in the area of asymmetric electrooxidation using chemically modified electrode was made by a successive work by Miller’s group in 1976. Using modified graphite electrodes, they successfully reported anodic oxidation of **38** to the corresponding sulfoxide **39** with excellent chemical yield although the value of enantiomeric excess was considerably low (Scheme 16) [46].

**Scheme 17:**
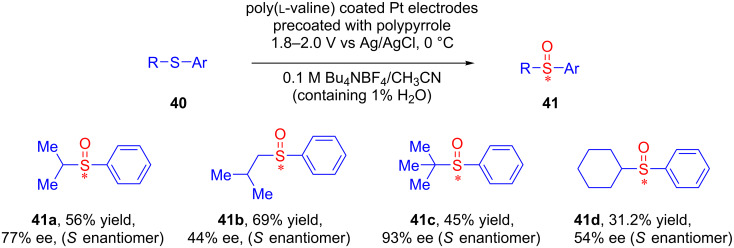
Asymmetric oxidation of unsymmetric sulfides using poly(amino acid)-coated electrodes.

A few years later, Komori and Nonaka, in two consecutive reports, established a modified method for the asymmetric electrochemical oxidation of alkyl aryl sulfides **40** to their corresponding sulfoxides **41** using poly(amino acid)-coated platinum electrodes with moderate yield and good to excellent enantiomeric excess (Scheme 17) [47–48].

**Scheme 18:**
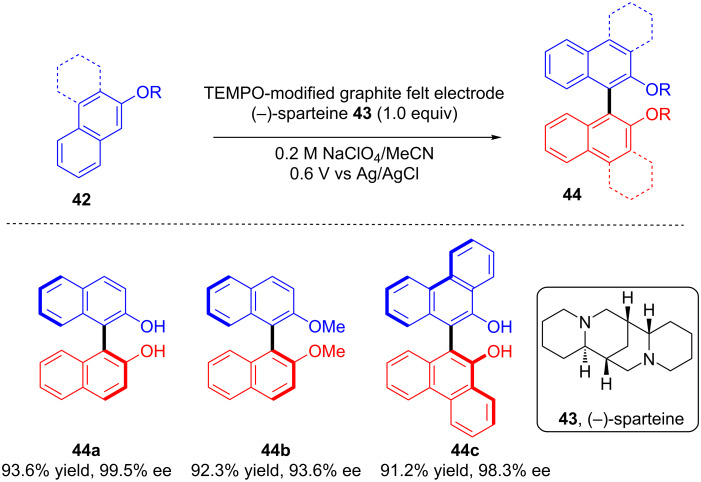
Enantioselective, electocatalytic oxidative coupling on TEMPO-modified graphite felt electrode in the presence of (−)-sparteine.

Osa and co-workers, in 1994, made an important contribution in the area by developing an enantioselective method for the electrocatalytic oxidative coupling of **42** using constant potential electrolysis of the substrates on a graphite felt electrode modified with TEMPO in the presence of (−)-sparteine **43**. The electrolysis resulted in (*S*)-binaphthyl type dimers **44** with excellent yield and enantiomeric excess (Scheme 18) [49].

**Scheme 19:**
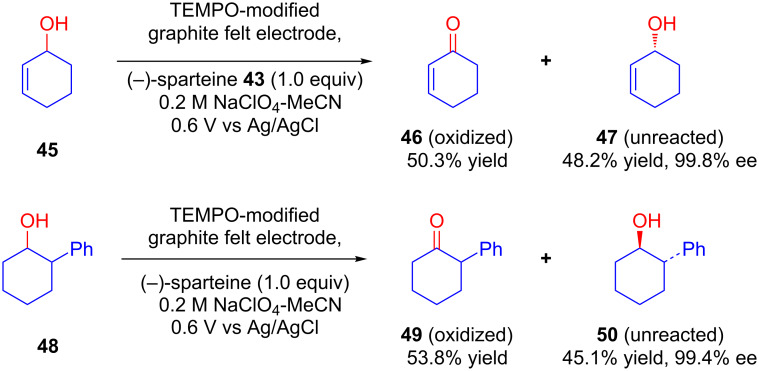
Asymmetric electrocatalytic oxidation of racemic alcohols on a TEMPO-modified graphite felt electrode in the presence of (−)-sparteine.

Later, the same group explored another application of TEMPO-modified graphite felt electrodes for enantioselective electrocatalytic oxidation of racemic secondary alcohols **45** and **48** (Scheme 19). (*S*)-Isomers of alcohol **48** possessing a chiral center at α-position to the hydroxy group were oxidized to the corresponding ketones **49** whereas (*R*)-**50** remained unreacted on a TEMPO-modified graphite felt electrode in the presence of (−)-sparteine. Enantiopurity of remaining alcohols were found to be more than 99% in all cases [50].

**Scheme 20:**
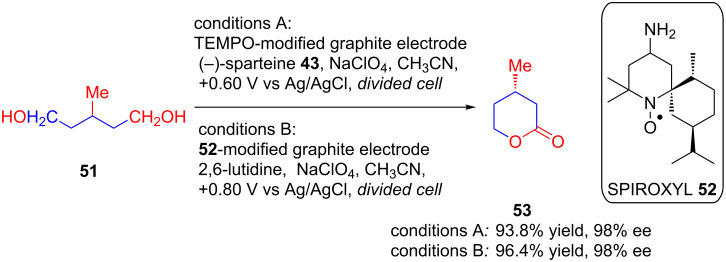
Asymmetric electrocatalytic lactonization of diols on TEMPO-modified graphite felt electrodes.

As a result of their study towards suitable modification of graphite felt electrodes for successful application in asymmetric electroorganic transformations, Kashiwagi’s group came up with two distinct protocols for asymmetric electrochemical lactonization of differently substituted diols. In 1996, they disclosed an asymmetric electrocatalytic method for lactonization of methyl-substituted diols **51** on a TEMPO-modified graphite felt electrode in the presence of the chiral base (−)-sparteine (**43**) with excellent enantioselectivity (conditions A, Scheme 20) [51]. Later in 2003, they reported another protocol for a graphite felt electrode for asymmetric electrochemical lactonization of diols **51**. However, in this method instead of a chiral base, they used 1-azaspiro[5.5]undecane *N*-oxyl **52** as a radical mediator for modifying the electrodes which resulted in optically active lactones **53** with enantiopurity of up to 99% (conditions B, Scheme 20) [52].

### Chiral medium

In this section, we present a compact overview of different categories of chiral media developed by organic chemists to induce asymmetry in electrochemical transformations. Chiral media can be further classified into subcategories such as chiral solvents, chiral electrolytes, chiral mediators, and chiral catalysts.

#### Chiral solvents

**Scheme 21:**
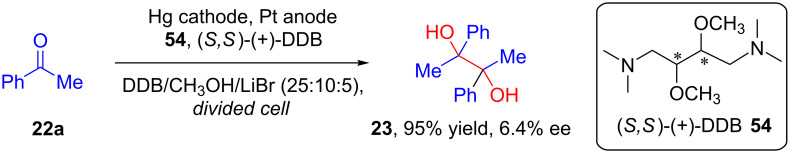
Asymmetric electrochemical pinacolization in a chiral solvent.

The only report on asymmetric electrochemical reaction in chiral solvent was published by Seebach and Oei in 1975 [53]. The asymmetric photodimerization of acetone to pinacol was conducted using amino-ether DDB **54** as a chiral solvent. Under the optimized reaction conditions, this electrolysis strategy allowed the electroreduction of acetophenone (**22a**) to pinacol (**23**) with 6.4% ee (Scheme 21). As mentioned by the author, the radical intermediate diffuses from the double layer, and dimerization occurs in the chiral solvent sphere in the solution phase.

#### Chiral supporting electrolyte

**Scheme 22:**
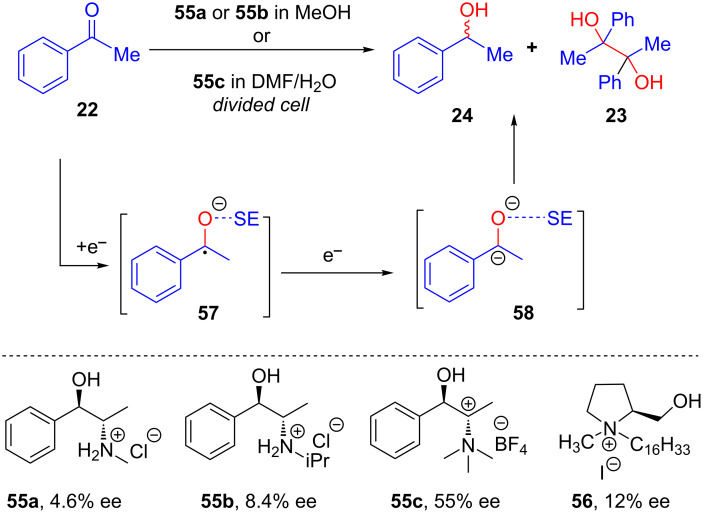
Asymmetric electroreduction using a chiral supporting electrolyte.

Electroorganic transformations using chiral supporting electrolytes have not been extensively explored, and the first example of such a reaction was reported by Horner in 1968 [54]. Interestingly, 4.6% ee was achieved in the electroreduction of acetophenone using ephedrine hydrochloride (**55a**) as chiral supporting electrolyte. A slight improvement in the enantioselectivity (8.4% ee) was observed using **55b** [55]. Kodama explained the reason of chiral induction in terms of ion pair interaction [56]. A further improvement in stereoselectivity was reported by Yadav, who added tetrabutylammonium trifluoroborate as an additional supporting electrolyte along with chiral **55c** (Scheme 22) [42,57].

**Scheme 23:**
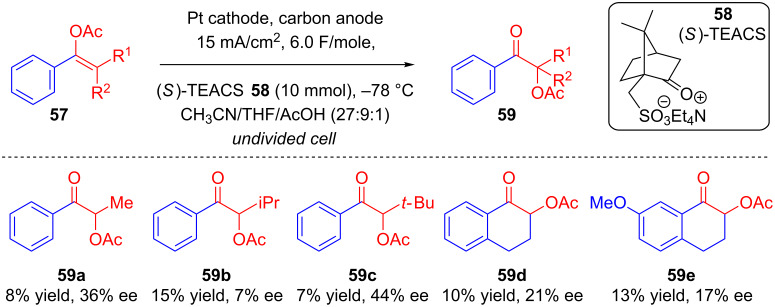
Asymmetric anodic oxidation of enol acetates using chiral supporting electrolytes.

In 2003, Nishiguchi’s group explored the anodic oxidation of enol acetates **57** upon constant current electrolysis in an undivided cell at −78 ºC in a mixture of solvents containing (*S*)-tetraethylammonium camphorsulfonate **58** as a chiral supporting electrolyte [58]. This resulted in the corresponding α-acetoxy ketones **59**, with low to moderate ee values (Scheme 23).

#### Chiral mediators

Chiral mediators are substances that allow or facilitate homogeneous electron transfer from the electrode to the substrate. This could be considered indirect electrolysis as the mediator takes an electron from the solid electrode and performs the organic transformation. This strategy has been shown to be suitable for inducing chirality, particularly in electro-oxidation reactions. Many chiral TEMPO-derived compounds have been explored as chiral mediators in electrochemistry. Notably, the distinction between mediators and catalysts can be difficult, but we have grouped them in terms of their role as per our classification.

**Scheme 24:**
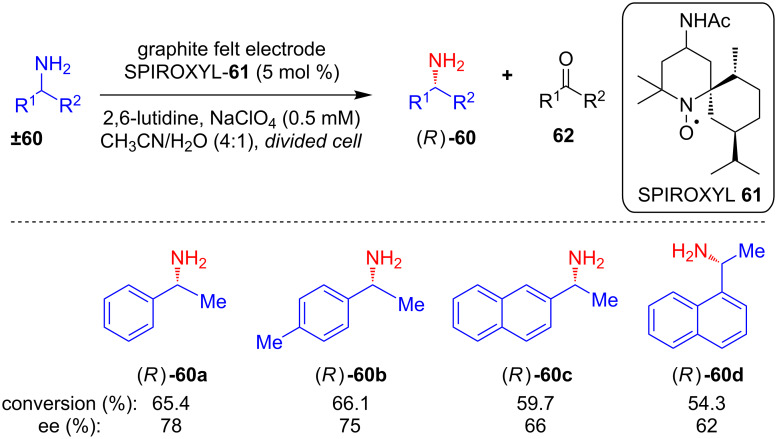
Kinetic resolution of primary amines using a chiral *N*-oxyl radical mediator.

In 1999, Kashiwagi and his group explored the applicability of chiral azaspiro-*N*-oxyl radical SPIROXYL (**61**) in the kinetic resolution of primary amines **60** via their electrocatalytic oxidation to the corresponding ketones **62**, and remaining amines (*R*)-**60** were obtained with very high enantioselectivity. The controlled potential electrolysis was carried out in a divided cell in the presence of a base and a catalytic amount of **61** (Scheme 24) [59]. A similar strategy using *N*-oxalyl radicals as chiral mediators has also been explored for the enantioselective electrocatalytic oxidation of secondary alcohols [60].

**Scheme 25:**
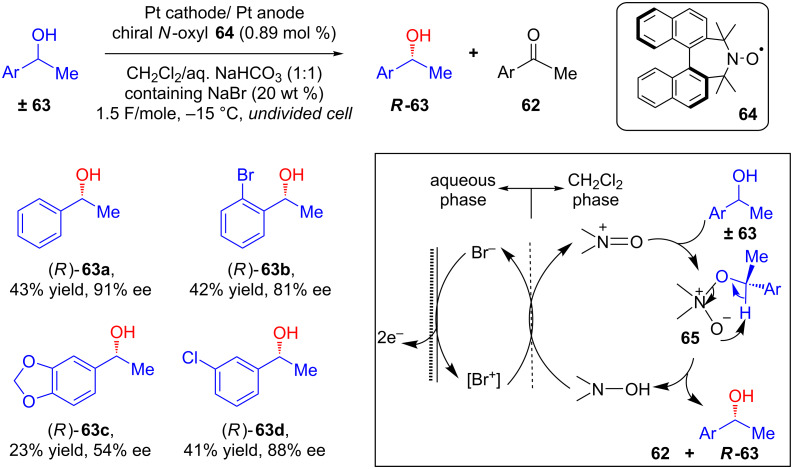
Chiral *N*-oxyl-radical-mediated kinetic resolution of secondary alcohols via electrochemical oxidation in a biphasic system.

Tanaka and co-workers subsequently published another article on the kinetic resolution of *sec*-alcohols **63** via electrochemical oxidation in an undivided cell under constant current [61]. This time, they used catalytic amount of a different chiral *N*-oxyl radical mediator **64** (Scheme 25). The authors proposed that in the biphasic CH_2_Cl_2_/water medium, [Br^+^] is initially generated from [Br^−^] by electrooxidation in the aqueous phase. The [Br^+^] thus generated oxidizes the *N*-oxyl/*N*-hydroxy species, leading to the formation of the *N*-oxoammonium species in CH_2_Cl_2_. The nucleophilic addition of racemic **63** to this *N*-oxoammonium species might further generate **65** and a steric hindered environment would favor the formation of (*S*)-**63** over (*R*)-**63**. Hence, (*S*)-**63** would be converted to ketone **62** via hydride transfer, whereas (*R*)-**63** might be predominantly retained.

**Scheme 26:**
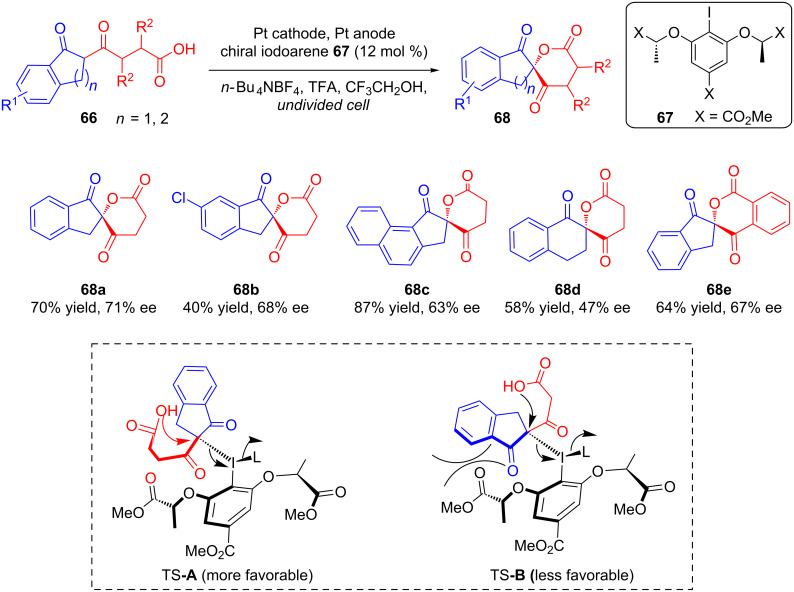
Chiral iodoarene-mediated asymmetric electrochemical lactonization.

Very recently, Wirth’s group demonstrated the use of chiral iodoarene **67** as a redox mediator for the electrochemical lactonization of diketo acid derivatives **66** (Scheme 26). Galvanostatic electrolysis of **66** in an undivided cell using a catalytic amount of **67** resulted in the corresponding lactones **68** in moderate yields and good stereoselectivities. The stereoselectivity was proposed to be controlled through the preferential formation of transition state **A** over **B** after reaction of the enolates of **66** with chiral iodoarene **67**. Steric factors restrict the formation of transition state **B**, and the reaction preferentially proceeds via transition state **A** to stereoselectively form the lactones [62].

#### Chiral catalysts

Achieving enantiocontrol in electrochemical transformations using various catalysts as chiral sources has been studied in greater detail than the other strategies mentioned in this review. As per our classification, we further categorize this class into metal catalysts, enzyme catalysts and organocatalysts.

**Scheme 27:**
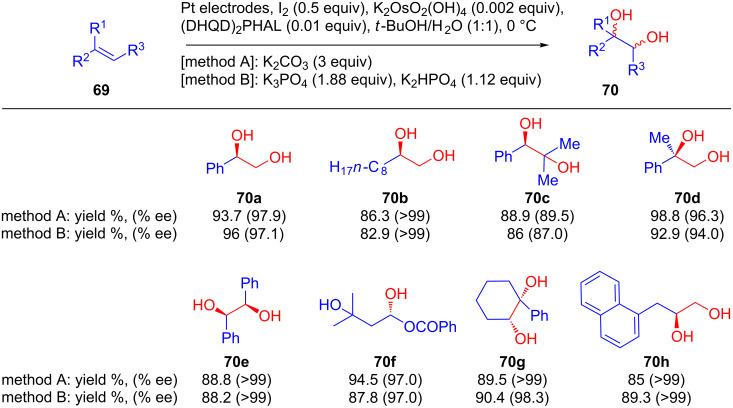
Os-catalyzed electrochemical asymmetric dihydroxylation of olefins using the Sharpless ligand and iodine as the oxidizing mediator.

**Metal catalysts:** In 1992, Amundsen and co-workers developed an electrochemical approach for a Sharpless asymmetric dihydrohydroxylation of alkenes using an osmium complex [63]. In this protocol, the traditional chemical oxidant was replaced by an anode that regenerates [FeCN)_6_]^3−^ via an Os(VIII) to Os(VI) interconversion. Similarly, Torri and co-workers investigated the asymmetric electrodihydroxylation of olefins **69** in an undivided cell under constant current using potassium osmate as a catalyst in presence of Sharpless ligand and using iodine as the oxidizing mediator (Scheme 27). Two sets of conditions were developed, and they differ in the inorganic bases used; method A involves K_2_CO_3_, whereas method B uses a mixture of K_3_PO_4_ and K_2_HPO_4_. However, olefins **69** were converted to the corresponding dihydroxylated products **70** in excellent yields as well as enantiomeric excesses regardless of the nature of the substituent or reaction conditions [64]. Further improvements of these methods have recently been achieved by Moeller et al. who designed a photovoltaic apparatus for the efficient Sharpless dihydroxylation of styrene in up to 94% enantiomeric excess [65].

**Scheme 28:**
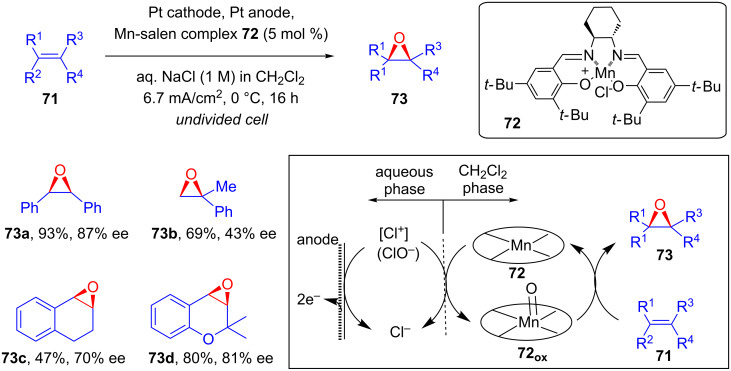
Asymmetric electrochemical epoxidation of olefins catalyzed by a chiral Mn-salen complex.

Tanaka and co-workers explored the catalytic applicability of optically active Mn-salen complex **72** towards asymmetric electroepoxidation of olefins **71** in a biphasic CH_2_Cl_2_/aqueous NaCl system (Scheme 28). The constant current epoxidation of **71** in an undivided cell resulted in chiral epoxides **73** in good yields and moderate enantioselectivities [66].

**Scheme 29:**
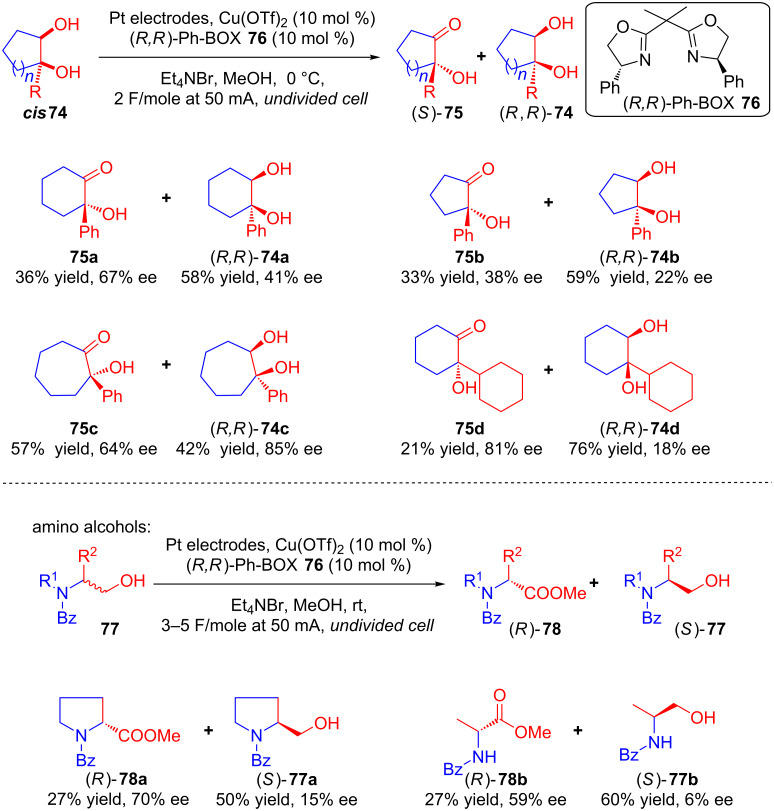
Asymmetric electrooxidation of 1,2-diols, and amino alcohols using a chiral copper catalyst.

In 2008, Onomura and co-workers described the electrochemical asymmetric oxidation of 1,2-diols **74** and amino alcohols **77** in presence of a Br^-^ mediator using Cu(OTf)_2_ and (*R,R*)-Ph-BOX **76** as the catalytic system (Scheme 29). The method enabled an asymmetric synthesis of α-hydroxycycloalkanones **75** and α-amino esters **78** along with the kinetic resolution of racemic **74** and **77**, respectively. The racemic substrates were electrolyzed in an undivided cell which resulted in the corresponding oxidized products and their kinetic resolution with good enantioselectivities [67].

**Scheme 30:**
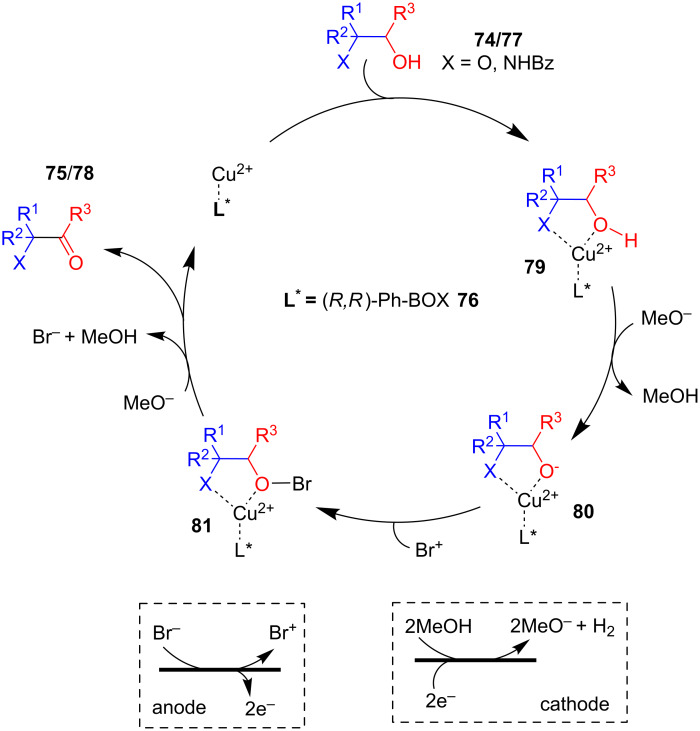
Mechanism of asymmetric electrooxidation of 1,2-diols, and amino alcohols using a chiral copper catalyst.

In the proposed mechanism, the substrate initially coordinates with the chiral Cu complex **79**. The complex **79** consisting of diols and a chiral copper catalyst Cu–L* easily gets deprotonated by cathodically generated MeO^−^ to afford alkoxide anions **80**, which reacts with anodically generated Br^+^ to form O-brominated intermediates **81**. Finally, MeO^−^ removed HBr from **81** to afford products **75**/**78** and regenerate the Cu–L* catalyst (Scheme 30).

**Scheme 31:**
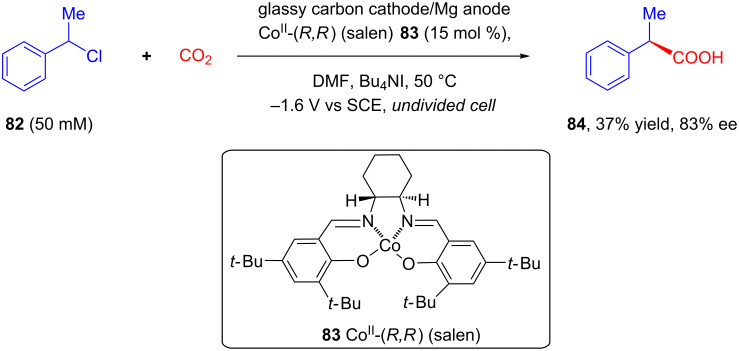
Enantioselective electrocarboxylation catalyzed by an electrogenerated chiral [Co^I^(salen)]^−^ complex.

In 2014, Wang and Lu reported a method for asymmetric fixation of CO_2_ via electrocarboxylation of **82** catalyzed by an electrogenerated chiral [Co^I^(salen)]^−^ complex [68]. The potentiostatic electrolysis of **82** in an undivided cell in presence of catalytic chiral Co(II)-(*R,R*)salen complex **83** in DMF saturated with CO_2_ resulted in the formation of **84** in a low yield but with good enantioselectivity (Scheme 31).

**Scheme 32:**
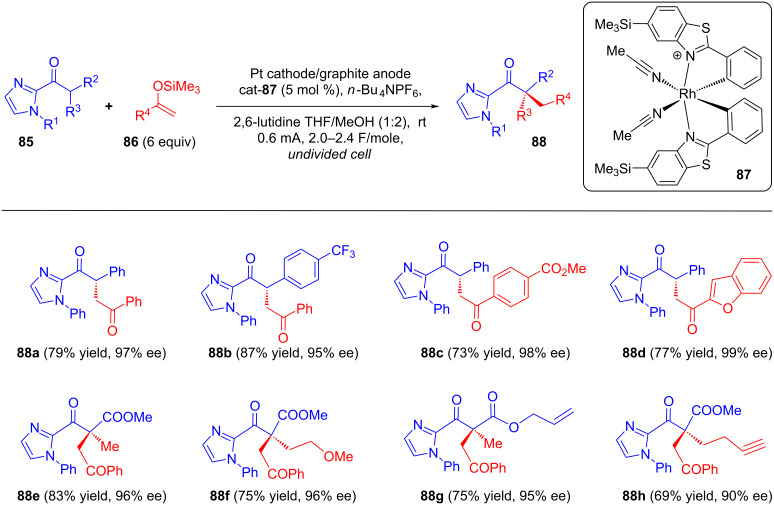
Asymmetric oxidative cross coupling of 2-acylimidazoles with silyl enol ethers.

Very recently, Meggers research group described an application of an asymmetric Lewis acid catalyst using electricity to provide a synthetic route towards chiral 1,4-dicarbonyls bearing tertiary and all-carbon quaternary stereocenters via oxidative cross coupling of 2-acylimidazoles **85** with silyl enol ethers **86** (Scheme 32). Chiral Rh complex **87** was exploited as a Lewis acid catalyst for the purpose of activating the substrate towards anodic oxidation by raising the energy of the HOMO upon enolate formation. Upon constant current electrolysis of a mixture of **85** and **86** in an undivided ElectraSyn 2.0 cell in presence of 2,6-lutidine as an external base and catalyst **87**, products with tertiary carbon stereocenters as well as all-carbon quaternary stereocenters **88** were obtained in good yields and enatioselectivities [69].

**Scheme 33:**
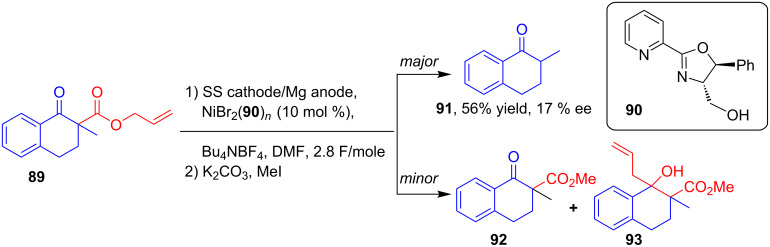
Ni-catalyzed asymmetric electroreductive cleavage of allylic β-keto ester **89**.

In 2002, Dunach’s group explored an electroreductive method for the cleavage of the allyl group of β-keto ester **89** followed by the transfer of the same to the carbonyl group to afford a mixture of **91** (major), **92** and **93** (Scheme 33). Constant current electrolysis of **89** in a single compartment cell using a sacrificial Mg anode was conducted in the presence of a Ni catalyst and chiral ligand **90**. After esterification and purification, **91** was isolated in a good yield and with moderate enantioselectivity [70].

**Scheme 34:**
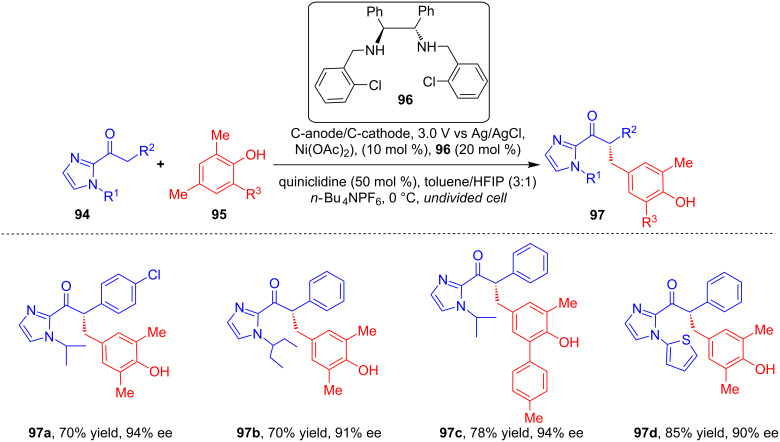
Asymmetric alkylation using a combination of electrosynthesis and a chiral Ni catalyst.

In another recent report, Guo and his group developed an electrochemical strategy for the asymmetric alkylation of 2-acylimidazole derivatives **94** with substituted *para*-methylphenols **95** using Ni(OAc)_2_ as a Lewis acid catalyst in presence of chiral diamine ligand **96** (Scheme 34) [71]. Based on their detailed mechanistic studies, the asymmetric induction was proposed to be realized via a combination of chiral Lewis acid-bound radical (generated through a single-electron anodic oxidation) and benzylic radical, generated through the anodic oxidation of **95**.

**Scheme 35:**
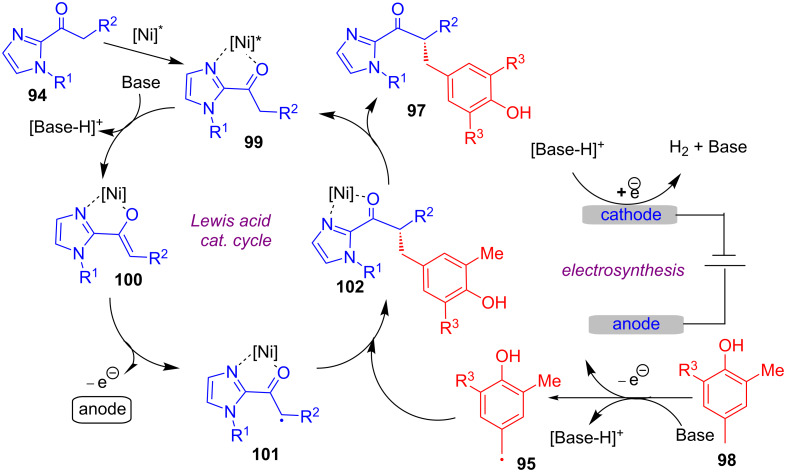
Mechanism of asymmetric alkylation using a combination of electrosynthesis and a chiral Ni catalyst.

As per the proposed catalytic cycle, initial coordination of the Lewis acid catalyst to 2-acylimidazole derivatives **94** generates the Lewis acid/enolate complex **100** upon deprotonation (Scheme 35). This is followed by the formation of intermediate **101** by electrolysis-induced SET oxidation. In a parallel electrochemical cycle, benzylic radical species **95** was delivered by the anodic oxidation. The radical–radical coupling reaction between **101** and **95** is proposed to afford the corresponding intermediate **102**. Then release of the Lewis acid catalyst gives the final product **97**. The stereocontrol step is related to the in situ generation of radicals with π-systems of chiral enamines and chiral enolates.

**Organocatalysts:** From the outset of electroorganic chemistry, chemists have devoted substantial effort towards applying organocatalysts in electroorganic synthesis. Recent advances integrating organocatalysis and electroorganic synthesis were elegantly presented by Boydston and Ogawa in their review article [72]. In this section, we will be presenting a concise description of the use of organocatalysts as chiral inductors in electroorganic synthesis.

**Scheme 36:**
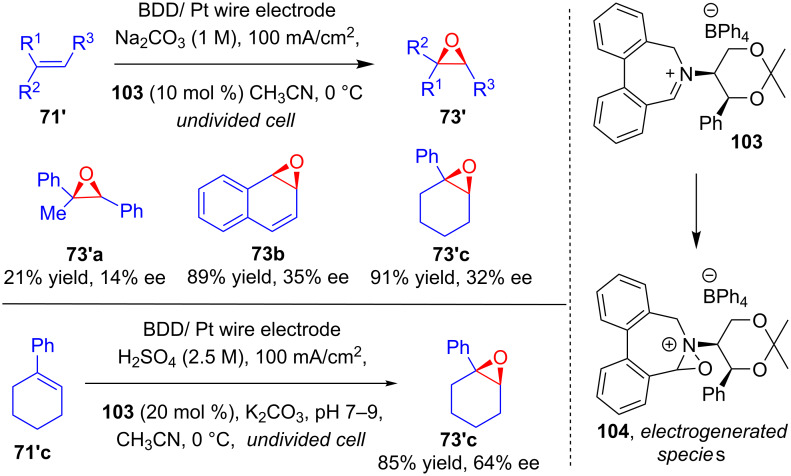
Asymmetric epoxidation by electrogenerated percarbonate and persulfate ions in the presence of chiral iminium salt.

In 2008, Page, Marken and their group reported a method for electricity-driven asymmetric organocatalytic epoxidation. The percarbonate oxidant required for this process was initially generated through anodic oxidation of aqueous Na_2_CO_3_ in an undivided cell under constant current electrolysis. A catalytic amount of chiral iminium salt **103** and olefinic substrate **71'** were added to the same reaction mixture, and chiral epoxides **73'** were obtained via oxidation by electrogenerated CO_4_^2−^ [73]. Moreover, they showed that electrogenerated persulfate generated in situ via a similar anodic oxidation of H_2_SO_4_ could act as an even better oxidant in presence of catalyst **103** to achieve the epoxidation of **71'c** with higher ee values (Scheme 36).

**Scheme 37:**
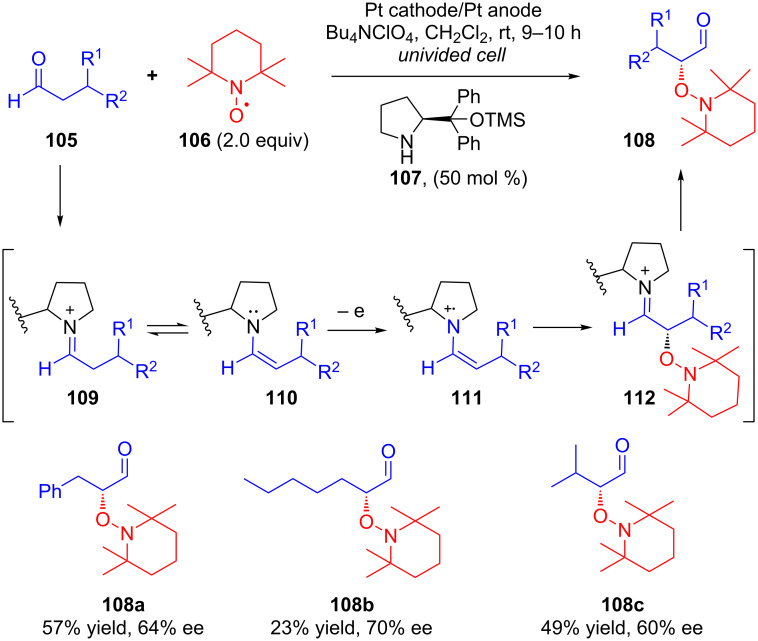
α-Oxyamination of aldehydes via anodic oxidation catalyzed by chiral secondary amines.

In 2009, Jang’s group disclosed the first example of the anodic oxidation of enamines (SOMO protocol) in terms of asymmetric α-oxyamination of aldehydes [74]. Galvanostatic electrolysis of aldehydes **105** in a single compartment cell using TEMPO (**106**) in presence of chiral secondary amine catalyst **107** resulted in the corresponding coupling products **108** in moderate yields and good enantioselectivities. After detailed electrochemical analysis, the authors proposed that the reaction proceeds through the intermediacy of radical cation **111**, generated via anodic oxidation of enamine **110** (Scheme 37).

**Scheme 38:**
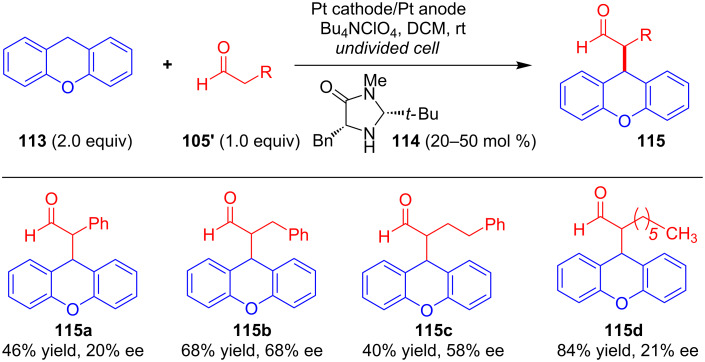
The α-alkylation of aldehydes via anodic oxidation catalyzed by chiral secondary amines.

The same group further extended the application of the SOMO protocol to the enantioselective α-alkylation of aldehydes [75]. Using modified chiral secondary amine **114**, the authors showed that constant current electrolysis of aldehydes **105'** with xanthenes **113** in an undivided cell resulted in α-alkylated aldehydes **115** with moderate enantioselectivity (Scheme 38).

**Scheme 39:**
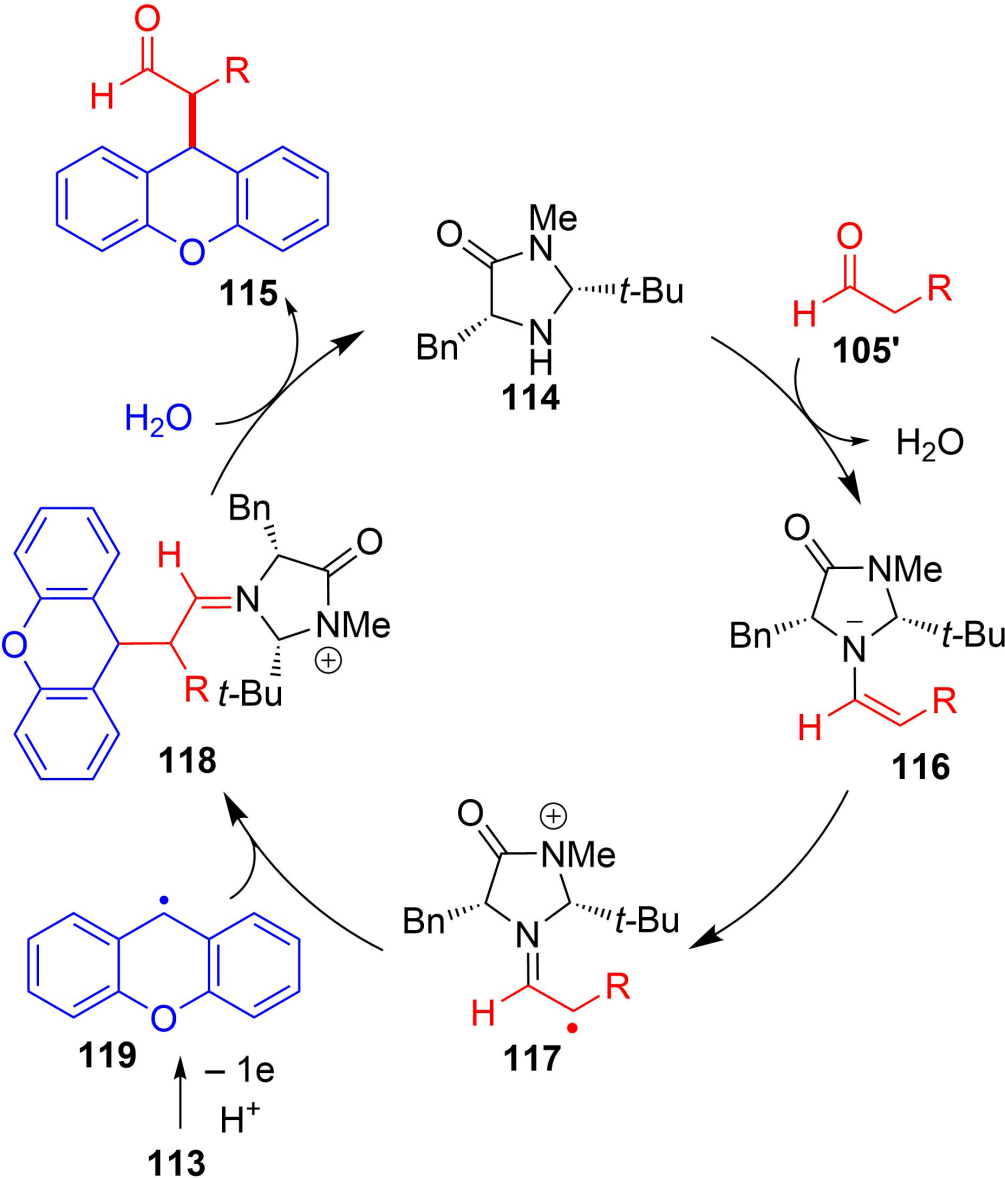
Mechanism of α-alkylation of aldehydes via anodic oxidation catalyzed by chiral secondary amines.

As shown in Scheme 39, the mechanism involved initial formation of radical cation **117** via anodic oxidation of enamine **116** (obtained from the condensation of **114** and **105'**), which then coupled with xanthene radical **119** (Scheme 39). Finally, hydrolysis of intermediate **118** takes place to obtained the final compound with regeneration of chiral catalyst **114**.

**Scheme 40:**
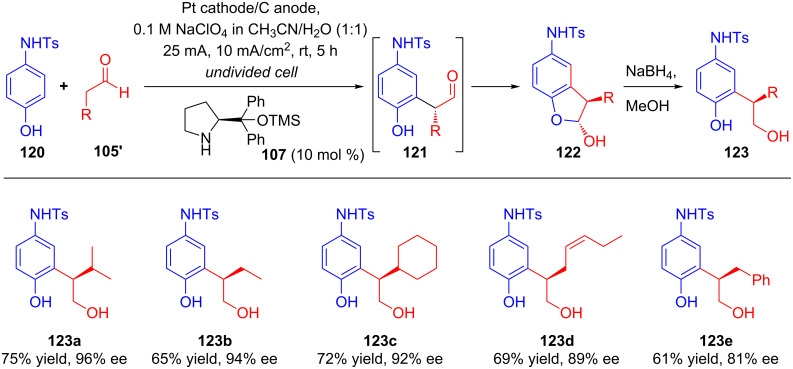
Electrochemical chiral secondary amine-catalyzed intermolecular α-arylation of aldehydes.

At almost the same time, Jorgensen et al. demonstrated that the intermolecular α-arylation of aldehydes via anodic oxidation could be performed using chiral secondary amine catalyst [76]. Constant current electrolysis of a mixture of aldehydes **105'** and *N*-protected aminophenol **120** in an undivided cell in the presence of catalyst **107** resulted in diastereomerically pure **122**, which were further reduced to the corresponding alcohol **123** and were isolated in good yields and excellent enantioselectivities (Scheme 40).

**Scheme 41:**
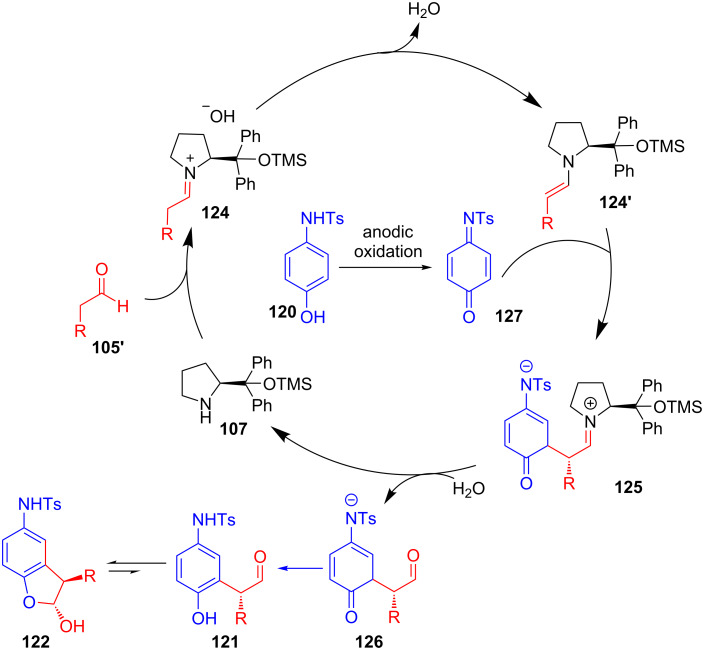
Mechanism of electrochemical chiral secondary amine-catalyzed intermolecular α-arylation of aldehydes.

As per the proposed mechanism, products **122** were obtained via nucleophilic addition of enamine **124'** to electrophilic intermediate **127** (generated from the anodic oxidation of **120**) followed by sequential hydrolysis, proton transfer and immediate intramolecular condensation (Scheme 41).

**Scheme 42:**
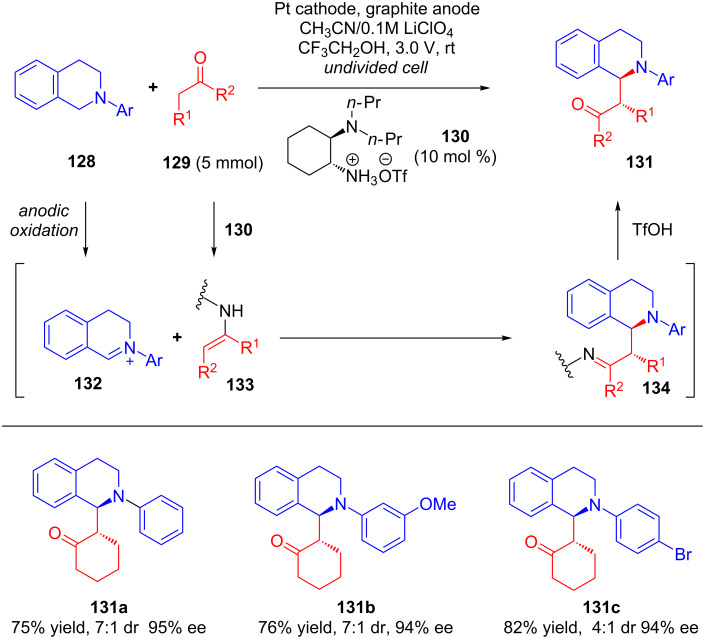
Asymmetric cross-dehydrogenative coupling of tertiary amines with simple ketones via an electrochemical oxidation catalyzed by chiral primary amines.

Very recently, Luo’s group disclosed another potential application of integrating electrochemical oxidation with chiral amine catalysis [77]. Using chiral primary amine **130** as a catalyst, the authors reported an electricity-driven cross-dehydrogenative coupling of ketones **129** with tertiary amines **128**. The potentiostatic electrolysis of a mixture of ketones **129** with tertiary amines **128** in an undivided cell under standard conditions furnished C1-alkylated tetrahydroisoquinolines **131** in high yields with excellent enantioselectivity (Scheme 42).

**Scheme 43:**
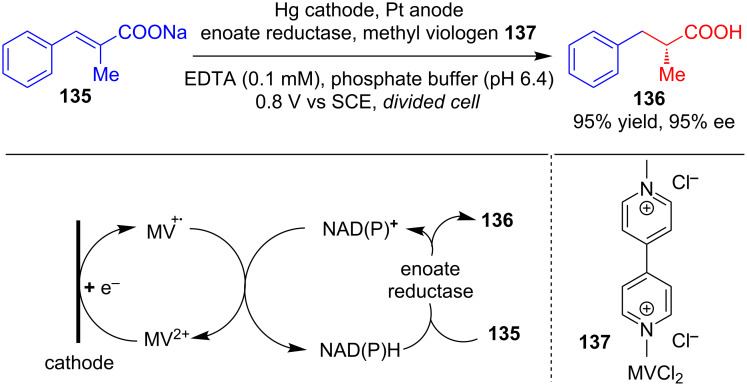
Electroenzymatic asymmetric reduction using enoate reductase.

**Enzyme catalysts:** In 1981, Tischer’s group reported the electroenzymatic asymmetric reduction of **135** using enolate reductase that afforded the corresponding chiral acid **136** in 95% yield and 95% ee. It has been shown that the cofactor NADH is oxidized during this process and can be regenerated using methyl viologen **137** as a reductive mediator (Scheme 43) [78].

**Scheme 44:**
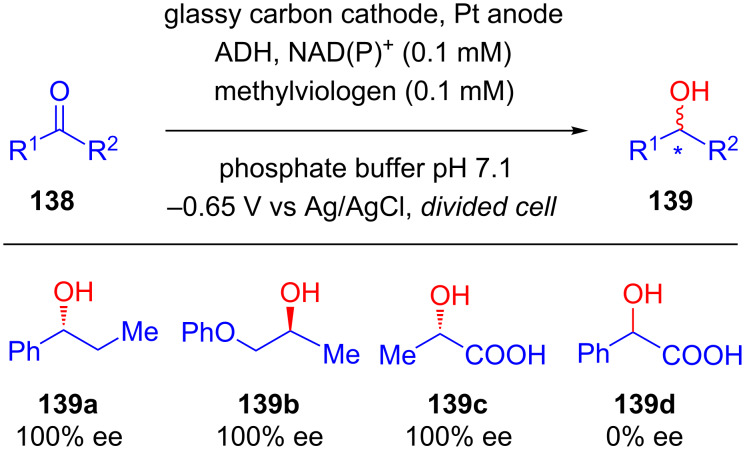
Assymetric reduction using alcohol dehydrogenase as the electrocatalyst.

In 1997, Yoneyama published a similar report on the asymmetric reduction of ketones **138** using alcohol dehydrogenase (ADH) as a catalyst [79]. In most cases, they achieved excellent stereocontrol; however, the method was ineffective in the case of phenyl ketoacid resulting in the formation of racemic alcohol **139d** (Scheme 44).

**Scheme 45:**
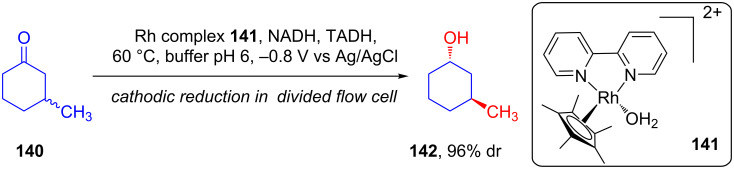
Asymmetric electroreduction catalyzed by thermophilic NAD-dependent alcohol dehydrogenase.

In 2007, Schmid explored the application of Rh complex **141** as a mediator for NADPH recycling [80]. This report showed that asymmetric electroreduction of cyclohexanone **140** in organic/aqueous media can be efficiently catalyzed by thermophilic NAD-dependent alcohol dehydrogenase (TADH) and result in the corresponding chiral alcohol **142** with a diastereomeric excess of 96% (Scheme 45).

**Scheme 46:**
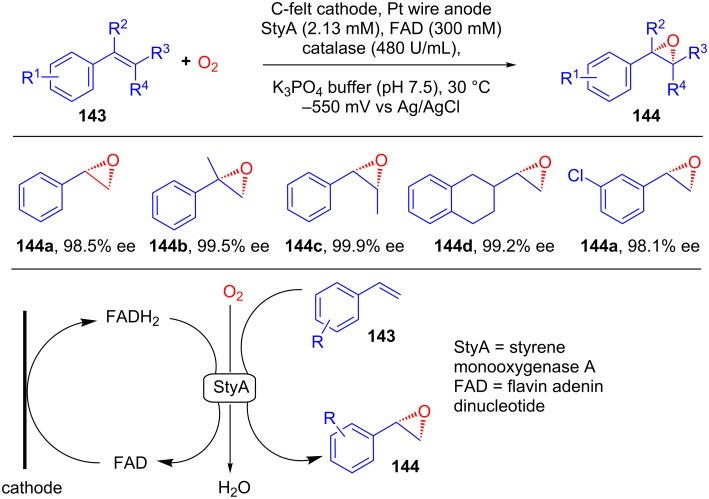
Asymmetric epoxidation of styrene by electrochemical regeneration of flavin-dependent monooxygenase.

The same group explored the practicality of direct electrochemical regeneration of a flavin-dependent monooxygenase as a catalyst for the asymmetric electrochemical epoxidation of styrenes [81]. They showed that upon potentiostatic electrolysis, styrene derivatives **143** could be transformed into their asymmetric epoxides **144** via direct electrochemical regeneration of FADH_2_. The authors claimed that this method is superior with respect to substitution of the complex native regeneration cycle, which consists of three enzymes (StyA, StyB, and an NADH-regenerating enzyme) and two cofactors (NADH and FAD), with only an oxygenase component and its flavin prosthetic group (Scheme 46).

**Scheme 47:**
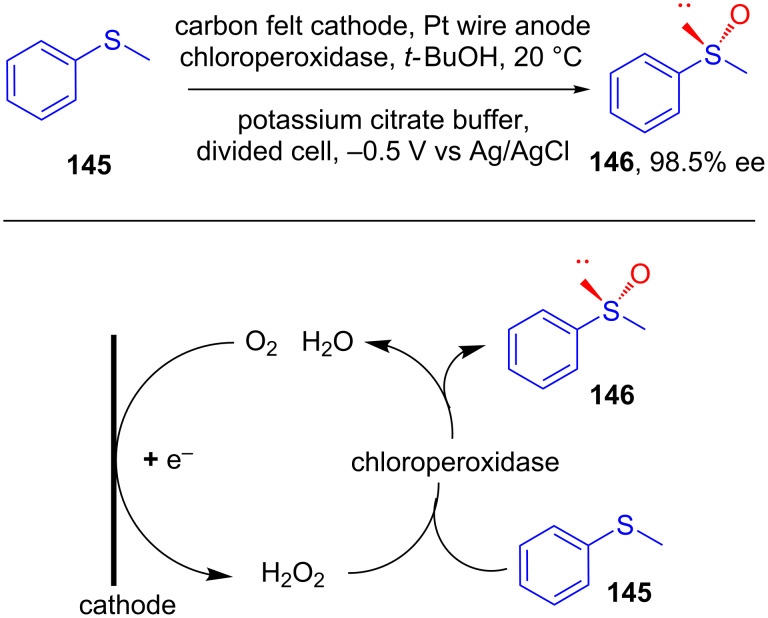
Asymmetric electroreduction using a chloroperoxidase catalyst.

In 2004, Liese reported the asymmetric sulfoxidation of thioanisole **145** with high productivity and excellent enantioselectivity using a chloroperoxidase catalyst. H_2_O_2_ generated in situ from the cathodic reduction of oxygen was proposed to be responsible for the enzyme-mediated thiol ether oxidation (Scheme 47) [82].

Vitamin B12-dependent enzymes are an exciting representative in the family of chiral inductors for electroorganic chemistry. These enzymes involve cobalt in the catalytically active center. In his article, Prof. Hisaeda reviewed vitamin B12-mediated electrochemical reactions in organic solvents in detail [83]. A number of pioneering results have been published in this area by leading electroorganic chemists [84–86].

**Scheme 48:**
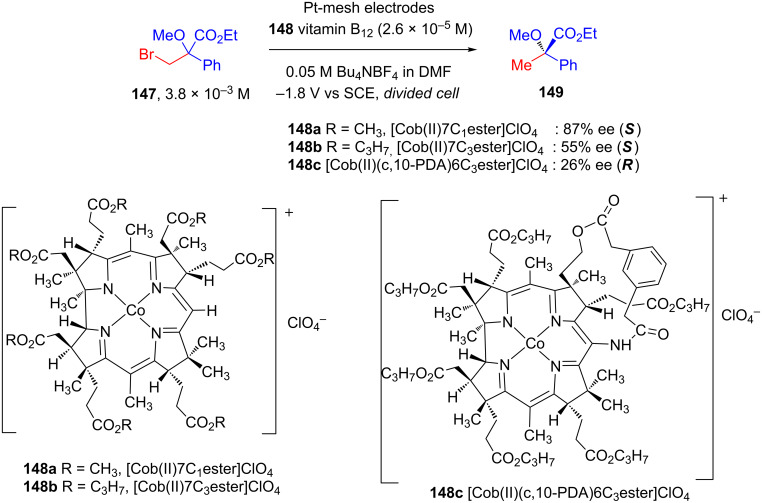
Asymmetric electrochemical transformation mediated by hydrophobic vitamin B12.

In 1994 and 1995, Murakami and his group published two sequential reports on the controlled potential electrolysis of racemic **147** catalyzed by a number of hydrophobic vitamin B12 enzymes **148** to afford hydrogen-substituted product **149** with good enantioselectivity (Scheme 48) [87–88].

### Chiral auxiliary approach

**Scheme 49:**
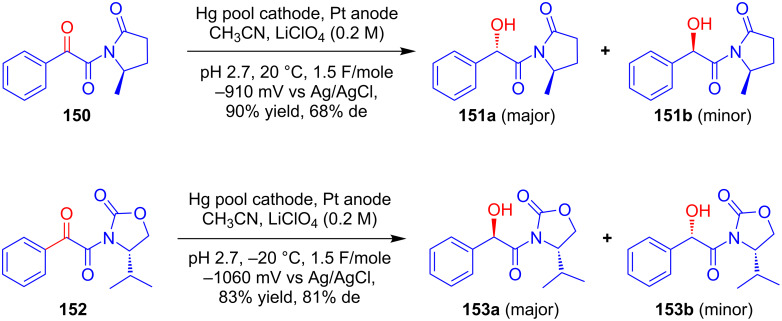
Diastereoselective cathodic reduction of phenylglyoxalic acids substituted with amines as chiral auxiliaries.

In 1994, Zielinski and Schäfer made a vital contribution in the field of asymmetric electrosynthesis in terms of the diastereoselective cathodic reduction of the carbonyl group of chiral phenylglyoxamides **150** and **152** [89–90]. After initial conversion to its corresponding amides using chiral auxiliaries phenylglyoxalic acid was further converted to mandelic acid derivatives **151** and **153**, respectively, upon reduction at a mercury pool cathode. The electrolysis afforded an excellent chemical yield along with moderate to good diastereomeric excesses. Moreover, the recovery of chiral auxiliaries upon hydrolysis of the major diastereomers of **151a** and **153a**, respectively, allowed access to optically active mandelic acid esters (Scheme 49).

**Scheme 50:**
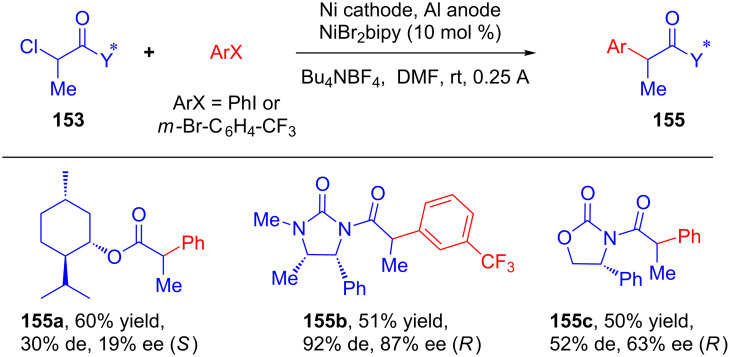
Ni-catalyzed asymmetric electroreductive cross coupling of aryl halides with α-chloropropanoic acid derivatives bearing chiral auxiliaries.

In 1997, Durandetti’s group published a Ni-catalyzed method for the electroreductive coupling between aryl halides and α-chloropropionic acid derivatives **153** in which the asymmetry was induced by chiral auxiliaries attached to the propionic acid derivatives [91]. Cross-coupling products **155** were obtained in satisfactory yields with good diastereomeric ratios. Moreover, the enantiomeric excess of products **155** were measured after their conversion to the corresponding esters or imides and no racemization was observed during the reaction (Scheme 50).

**Scheme 51:**
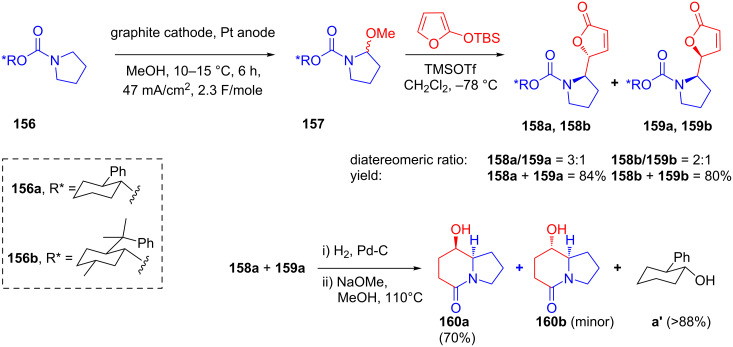
Electrochemical Mannich addition of silyloxyfuran to in situ-generated *N*-acyliminium ions.

In 2000, Pilli and co-workers published a vinylogous Mannich addition of silyloxyfuran to chiral *N*-acyliminium ions generated in situ from **157** which had been obtained from the anodic oxidation of **156** bearing a cyclohexyl-based chiral auxiliary ([92]. The authors established that the Mannich addition occurred exclusively on the *Si*-face of the *N*-acyliminium ions, resulting in the *threo*-isomer as the major isomer (with moderate yields and good diastereomeric ratios). Upon catalytic hydrogenation followed by methanolysis, *threo*-**158a**, and **159a** were further converted to the corresponding lactams **160a** and **160b** enabling efficient recovery of chiral auxiliaries (Scheme 51). Furthermore, the same group reported an identical method for the TiCl_4_-promoted addition of allyltrimethylsilane to *N*-acyliminium ions containing the same cyclohexyl-based chiral auxiliaries [93].

**Scheme 52:**
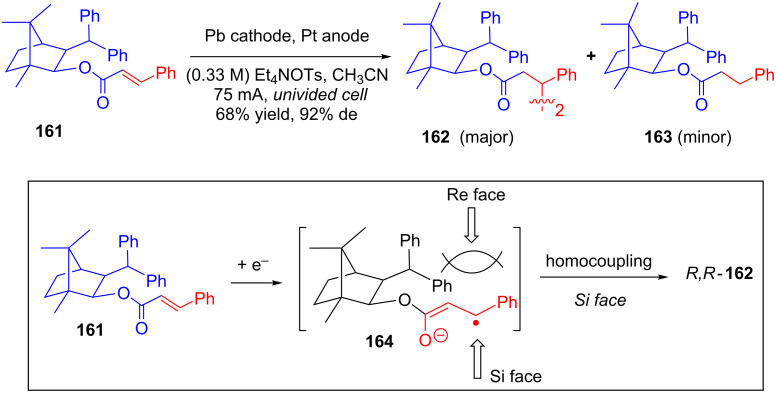
Stereoselective electroreductive homodimerization of cinnamates attached to a camphor-derived chiral auxiliary.

While studying the effect of chiral auxiliaries on the enantioselective electroreduction of cinnamic acid derivatives, which resulted in the corresponding hydrodimers, Kise and his group investigated a number of chiral auxiliaries and found that chiral auxiliary derived from (+)-camphor was the most effective one for asymmetric electroreductive hydrocoupling of cinnamates **161** [94–95]. The constant current electrolysis of **161** in an undivided cell resulted in acyclic homodimer **162** (with good yield and excellent diastereoselectivity) along with a certain percentage of **163**. The chiral auxiliary was released almost quantitatively after LAH reduction of **162**. The authors proposed a hydrocoupling mechanism involving the one-electron reduction of **161** to generate radical anion **164** followed by the coupling of another radical anion from the *Si*-face (Scheme 52).

**Scheme 53:**
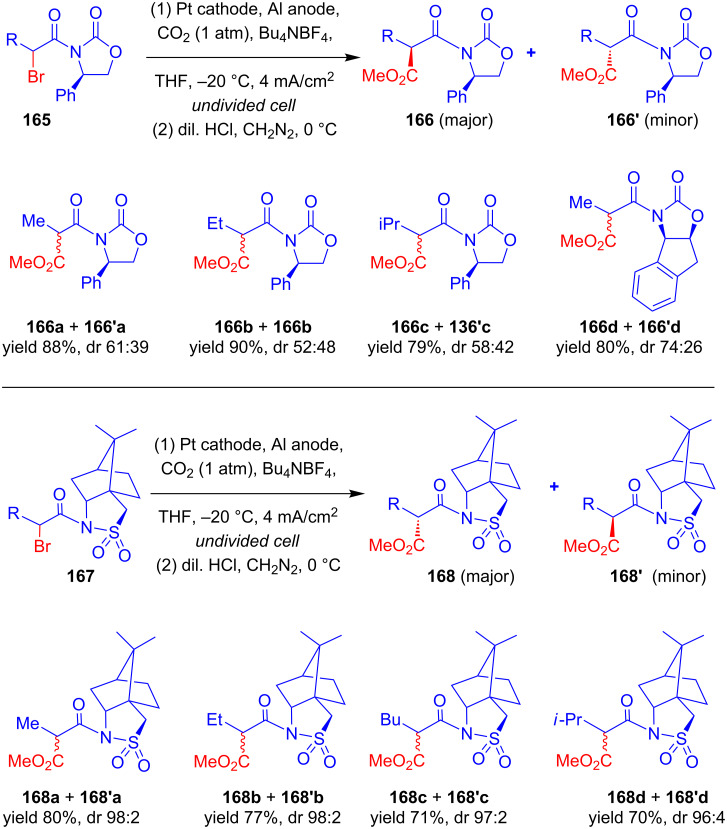
Diastereoselective electrochemical carboxylation of chiral α-bromocarboxylic acid derivatives.

In two sequential reports, Feroci and Inesi discussed the electrochemical carboxylation of chiral α-bromocarboxylic acid derivatives **165** substituted with Evans-type chiral auxiliaries [96–97]. The cathodic reduction of the C–Br bond in presence of CO_2_ followed by treatment with diazomethane resulted in the corresponding malonic ester derivatives **166**, and the diastereomeric ratio was greatly affected by various electrochemical conditions, and higher yields (88%) were obtained at the expense of diastereoselectivity (dr 61:39) [**166a** + **166'a**]. The authors next modified the chiral auxiliary and obtained the best results with α-bromocarboxylic acid derivatives **167** substituted with Oppolzer’s camphor sultam, which provided carboxylated products **168** in good yield (80%) and excellent diastereomeric ratio (98:2) (Scheme 53).

**Scheme 54:**
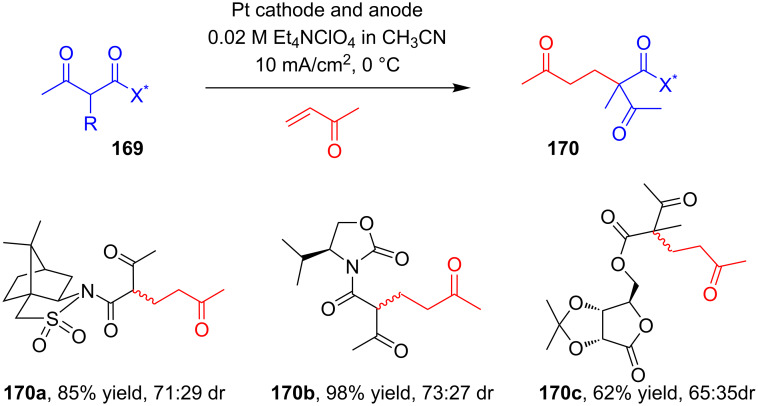
Electrocatalytic stereoselective conjugate addition of chiral β-dicarbonyl compounds to methyl vinyl ketone.

The same group reported an electrocatalytic Michael addition of chiral acetoacetic derivatives **169** substituted with chiral auxiliaries to methyl vinyl ketone for stereoselective construction of the quaternary carbon centers in **170** (Scheme 54). While screening a number of chiral auxiliaries, the authors found that upon electrolysis under galvanostatic conditions at low temperature, the acetoacetates of Oppolzer’s sultam and Evans’ oxazolidin-2-ones afforded the best yields and diastereomeric ratios of addition products **170** relative to what was achieved with other chiral auxiliaries [98].

**Scheme 55:**
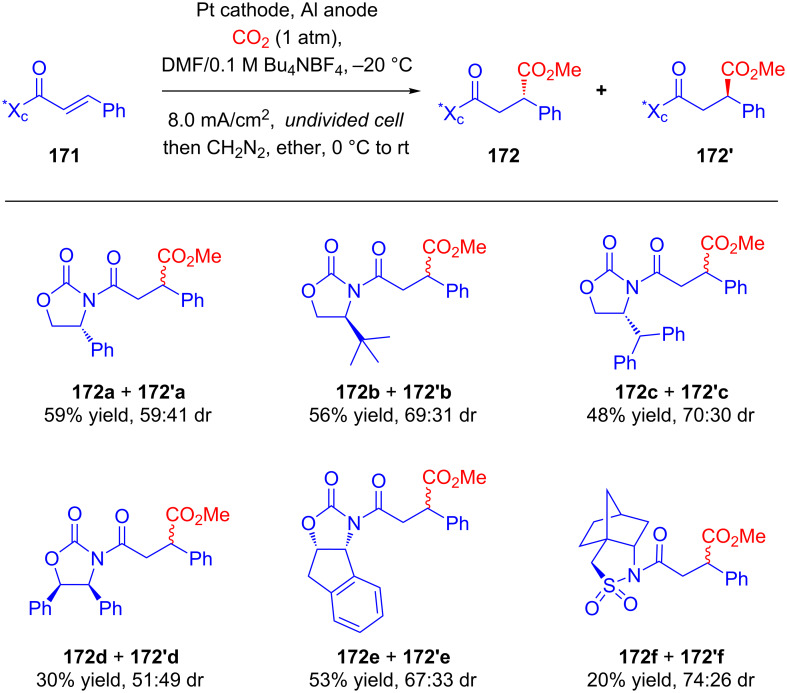
Stereoselective electrochemical carboxylation of chiral cinnamic acid derivatives under a CO_2_ atmosphere.

As a part of studies on the effects of chiral auxiliaries on the stereoselectivity in electrochemical transformations, Feroci and Inesi developed a stereoselective carboxylation of cinnamic acid derivatives **171** substituted with chiral auxiliaries [99]. The substrate **171** was subjected to galvanostatic reduction under CO_2_ atmosphere in an undivided cell. Carboxylation followed by treatment with diazomethane resulted in the corresponding 2-phenylsuccinate esters **172** and **172'** in satisfactory yields. The best diastereoselectivities were obtained using Oppolzer’s camphor sultam (**172f** + **172f'**) and 4*R*-(diphenylmethyl)oxazolidin-2-one (**172c** + **172'c**) as chiral auxiliaries (Scheme 55).

**Scheme 56:**
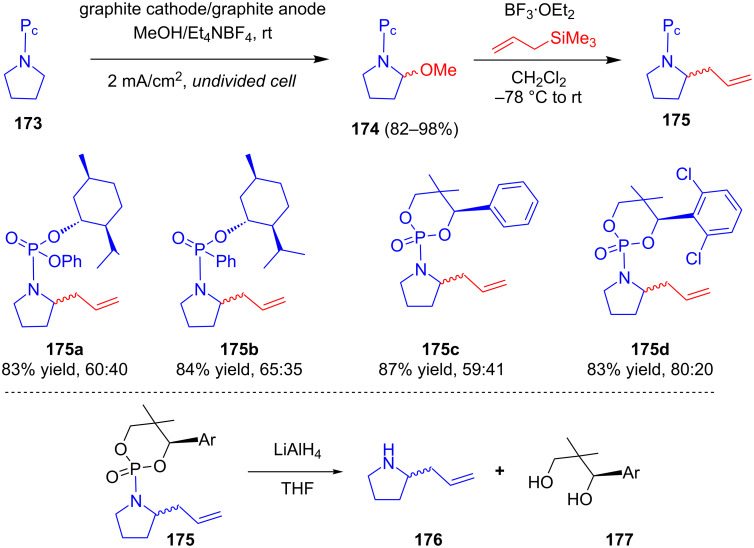
Electrochemical diastereoselective α-alkylation of pyrrolidines attached with phosphorus-derived chiral auxiliaries.

Martens and his group synthesized chiral compounds based on phosphorus esters and investigated their effects as chiral auxiliaries in the α-alkylation of secondary amines via anodic oxidation [100]. The constant current methoxylation of *N*-protected chiral pyrrolidines **173** in an undivided cell resulted in **174** in excellent yield. Further nucleophilic substitution of **174** with allyltrimethylsilane in the presence of a Lewis acid afforded α-alkylated products **175**. The highest de was obtained in the case of **175d**. The deprotection of **175** with LAH enabled access to α-alkylated pyrrolidines **176** with the release of **177** (Scheme 56).

**Scheme 57:**
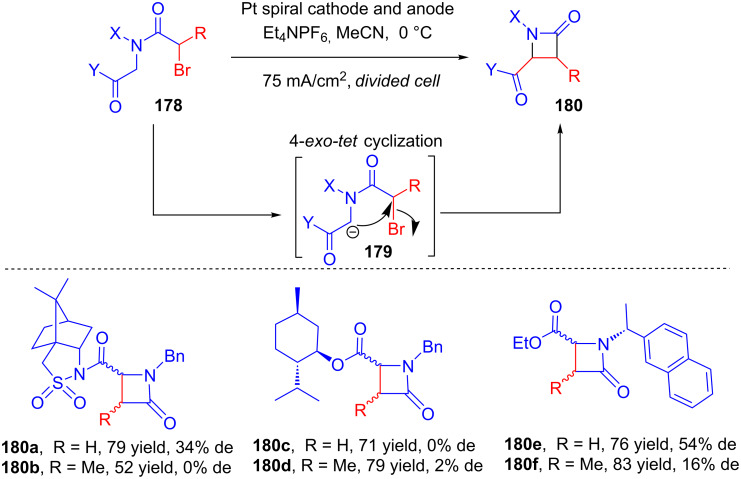
Electrogenerated cyanomethyl anion-induced synthesis of chiral *cis*-β-lactams from amides bearing chiral auxiliaries.

In 2007, Feroci disclosed an electrochemical strategy for the *cis*-stereoselective synthesis of chiral β-lactams **180** via a 4-*exo-tet* cyclization of bromo amides **178** with an acidic methylene group and bearing a chiral auxiliary [101]. The cyclization occurred via deprotonation of the acidic methylene group by a base (cyanomethyl anion) obtained from the galvanostatic reduction of acetonitrile/tetraethylammonium hexafluorophosphate (Scheme 57).

**Scheme 58:**
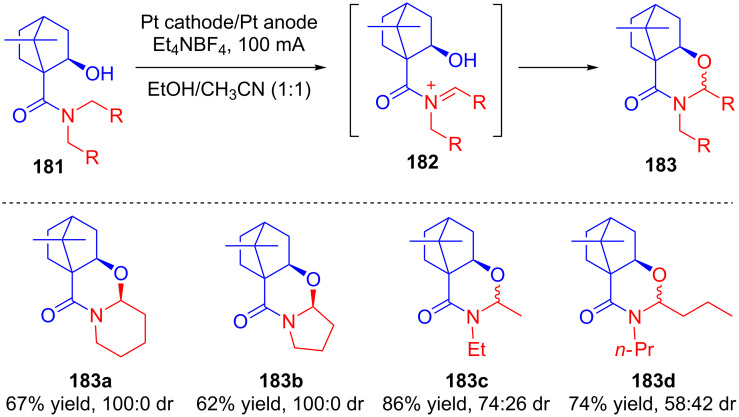
Diastereoselective anodic oxidation followed by intramolecular cyclization of ω-hydroxyl amides bearing isoborneol-based chiral auxiliaries.

Two years later, Lee and co-workers explored the efficacy of isoborneol-based chiral auxiliaries for asymmetric induction in intramolecular anodic oxidations of ω-hydroxyl amides [102]. Upon constant current electrolysis, substrates **181** were oxidized to **182**, which underwent in situ cyclization by intramolecular nucleophilic hydroxy group addition to afford a diastereomeric mixture of **183**. The study also revealed that when cyclic amines were used as substrates (**181a**, **181b**), the products were formed with 100% diastereoselectivity (**183a** and **183b**), whereas acyclic amines resulted in lower diastereoselectivity (**183c** and **183d**) even though the intramolecular attack of the hydroxy group to the iminium bond in **182** preferentially occurred from the *Si*-face over the *Re*-face in both cases (Scheme 58).

**Scheme 59:**
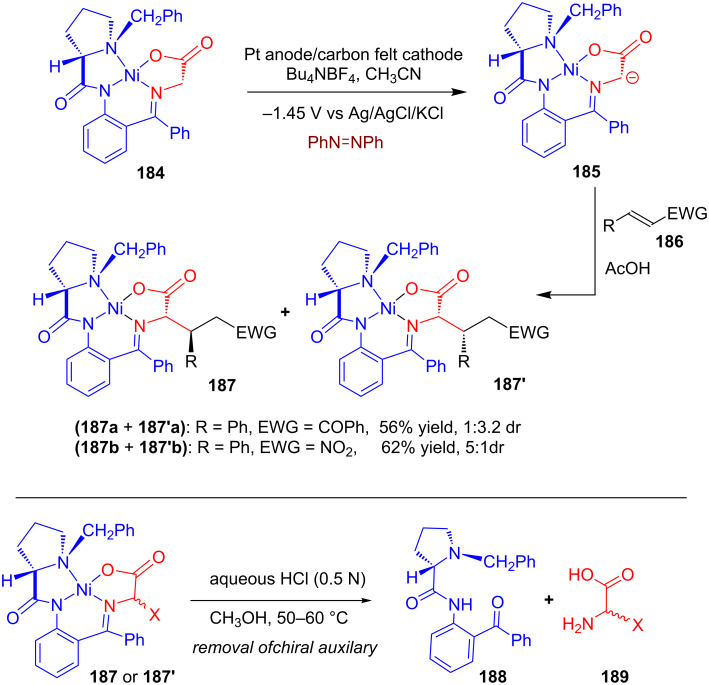
Electrochemical deprotonation of Ni(II) glycinate containing (*S*)-BPB as a chiral auxiliary: diastereoselective nucleophilic addition to Michael acceptors.

Magdesieva and his group developed an electrochemical method for the deprotonation of a Ni(II) glycinate complex containing (*S*)-*o*-[*N*-(*N*-benzylprolyl)amino]benzophenone [(*S*)-BPB] **188** as an chiral auxiliary moiety and explored its applicability in diastereoselective Michael addition reactions [103–104]. The glycine–nickel complex **184** was deprotonated using a radical anion generated from the electrochemical reduction of azobenzene. The anionic Ni complex **185** acted as a good *C*-nucleophile towards Michael acceptors **186** resulting in diastereoisomeric mixtures of **187** and **187'**. Moreover, the modified Ni-complexes obtained after Michael addition could be easily decomposed using HCl in MeOH, releasing functionalized amino acid **189** along with the quantitative recovery of the chiral auxiliary (*S*)-BPB **188** (Scheme 59).

**Scheme 60:**
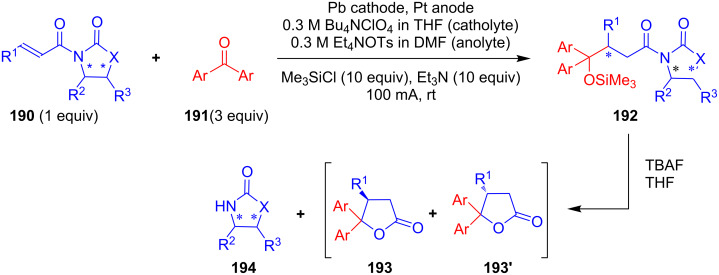
Enantioselective electroreductive coupling of diaryl ketones with α,β-unsaturated carbonyl compounds containing chiral auxiliaries.

Recently, Kise’s group reported an efficient method for the enantioselective synthesis of 4,5,5-trisubstituted γ-butyrolactones **193** using an electroreductive coupling of diaryl ketones **191** with α,β-unsaturated carbonyl compounds **190** bearing chiral auxiliaries derived from imidazolidin-2-one and oxazolidine-2-ones (Scheme 60). Compound **191** underwent 2e^−^ reduction under electrochemical conditions followed by *O*-silylation and afforded **192** with high diastereoselectivity. Further treatment of **192** with TBAF resulted in highly substituted optically active **193** with concomitant release of chiral auxiliary **194** (Table 1) [105].

**Table 1:** Enantioselective electroreductive coupling of diaryl ketones with α,β-unsaturated carbonyl compounds containing chiral auxiliaries.

**191**	**190**	**193**	Yield (%)	ee (%) **193**

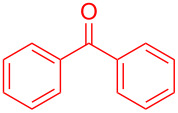 **191a**	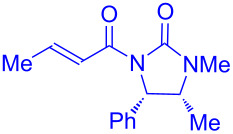 **190a**	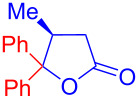 **193a**	63	99
	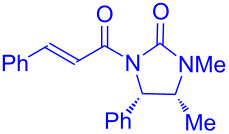 **190b**	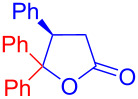 **193b**	60	93
	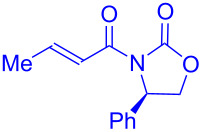 **190c**	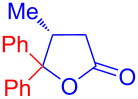 **193’a**	68	95
	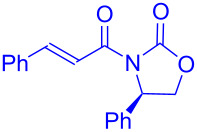 **190d**	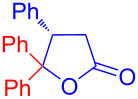 **193’b**	47	99
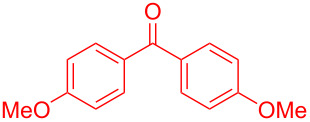 **191b**	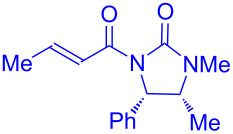 **190a**	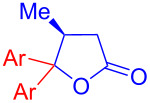 **193c** Ar = *p*-OMeC_6_H_4_	56	99
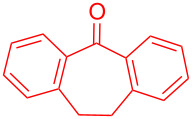 **191c**	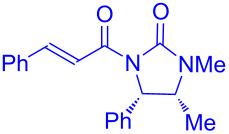 **190b**	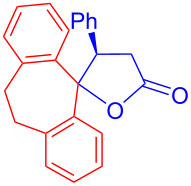 **193d**	67	93

### Application in the synthesis of natural products and bio-relevant compounds

Application of organo-electrosynthesis in complex molecule synthesis has recently been reviewed by Lundberg and Kärkäs [106]. In the following section of this review, we describe potential practical applications of asymmetric electroorganic reactions in the synthesis of different complex and biologically relevant compounds as well as natural products.

**Scheme 61:**
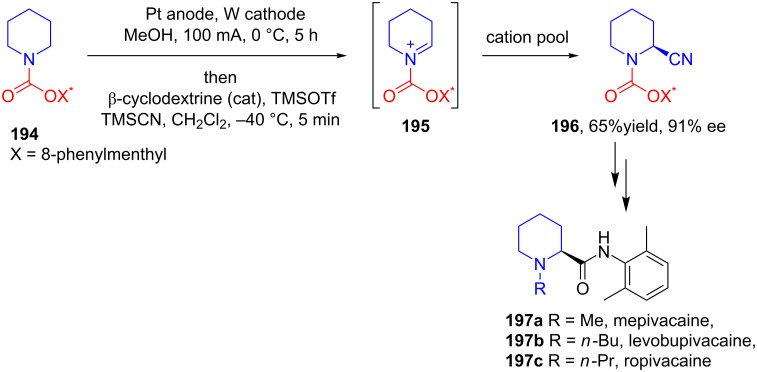
Asymmetric total synthesis of ropivacaine and its analogues using a electroorganic reaction as a key step.

In 2008, Pilli and Santos developed an innovative method for the enantioselective total synthesis of the local anesthetic drug ropivacaine (**197c**) and its analogues levobupivacaine (**197b**) and mepivacaine (**197a**) [107]. The key steps in the synthesis involved the initial anodic oxidation of cyclic *N*-carbamate **194** bearing an 8-phenylmenthyl group as a chiral auxiliary which generates in situ *N*-acyliminium ion **195** and this **195** upon reaction with nucleophilic CN^−^ in presence of catalytic TMSOTf and β-cyclodextrin as the cocatalyst provide **196** in a good yield and excellent stereoselectivity (Scheme 61).

**Scheme 62:**
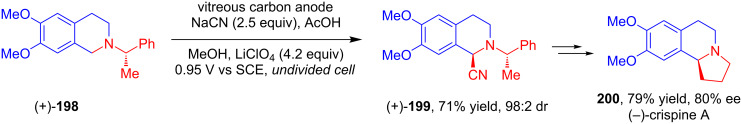
Asymmetric total synthesis of (−)-crispine A and its natural enantiomer via anodic cyanation of tetrahydroisoquinoline.

In 2011, Hurvois and his group reported a stereoselective electrochemical total synthesis of the tetrahydroisoquinoline alkaloid (−)-crispine A (**200**) and its natural enantiomer [108]. The initial steps involved the synthesis of precursor (+)**-198** from phenylacetic acid and (−)-α-PEA via sequential amidation, reduction and Pictet–Spengler cyclization reactions. The key anodic oxidation in the presence of NaCN converted (+)-**198** to α-amino nitrile (+)-**199** with excellent diastereoselectivity. Upon further alkylation, reduction and catalytic hydrogenolysis, (+)-**199** was converted to desired product (−)-**200** with 80% ee (Scheme 62).

**Scheme 63:**
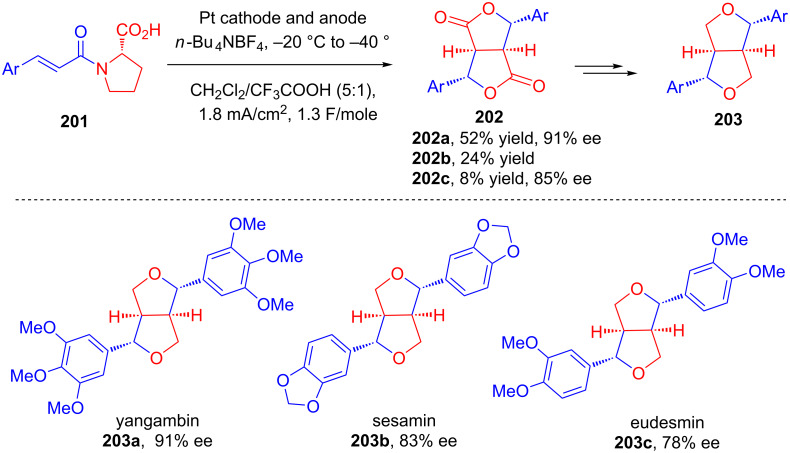
Asymmetric oxidative electrodimerization of cinnamic acid derivatives as key step for the synthesis of furofuran lignans.

An electrochemical method for the asymmetric oxidative dimerization of cinnamic acid derivatives was developed by Watanabe in 2016 [109]. The substrates for the electrochemical oxidation **201** were prepared from the corresponding cinnamic acids via condensation with ʟ-proline *tert*-butyl ester followed by ester hydrolysis. Upon oxidative dimerization under Ronlan’s electrochemical conditions, substrates **201** were converted to bislactones **202** with good enantioselectivity. The authors extended their method towards the asymmetric syntheses of furofuran lignans **203** from chiral bislactones **202**. Furofuran lignans **203** were synthesized in good to excellent enantioselectivity (Scheme 63).

## Conclusion and Future Directions

Extensive studies of electroorganic reactions have opened alternative pathways for synthetic transformations that are otherwise quite challenging. Not only the environmental advantages with respect to substituting toxic chemical reagents with mass-free electrons and minimizing the reagent waste generated, but also in terms of shortening of tedious multistep reaction sequences, these strategies enable advancements in reaction scalability as well as tunability.

Having described the research that has been conducted to achieve suitable ‘electrical’ replacements of conventional ‘chemical’ organic transformations, it is clear that the establishment of stereoselective variants of these synthetic operations remains challenging, making the use of electrochemistry to achieve stereoselective transformations an often unsolved problem. Modern synthetic laboratories have, however, made advances in this regard due to arduous studies resulting in a number of landmark publications with successful stereoselective electroorganic transformations. This review sought to compile significant accomplishments in this area and will hopefully serve as a handbook for practicing organic chemists and inspire them to focus on this relatively underdeveloped area of synthetic organic chemistry. Several challenges need to be addressed in order to see widespread application. While recent advancements in electrochemistry have been promising, readily available parallel screening technologies similar to those in homogeneous, heterogeneous, photo- as well as biocatalysis need to be developed. Parallel electrochemical reaction set-ups will, without doubt, accelerate further developments in the field of asymmetric electrochemistry and electrocatalysis. So far, most of the electrochemical reaction set-ups in this area rely on the use of less favorable electrodes, including Pt, Hg or Pb electrodes. Thus, more economical and ecological alternatives need to be examined, in particular if larger scale production is envisaged. Regarding stereochemical induction, the design of chiral media, similar to chiral solvents in asymmetric catalysis, is still limited. While the chemical modification of electrodes for the generation of chiral surfaces has shown some success, it seems that the most promising area in the further development of asymmetric electrochemistry and electrocatalysis will rely on the use of chiral mediators and chiral catalysts. Given the almost unlimited variations in combining electrochemistry with photo-, organo-, bio- and metal catalysts, many new and unexpected asymmetric reactions will be developed resulting in the further development of catalysis and synthesis in general.
